# Efficacy and Safety of Trifluridine/Tipiracil-Containing Combinations in Colorectal Cancer and Other Advanced Solid Tumors: A Systematic Review

**DOI:** 10.1093/oncolo/oyae007

**Published:** 2024-02-16

**Authors:** Kohei Shitara, Alfred Falcone, Marwan G Fakih, Ben George, Raghav Sundar, Sandip Ranjan, Eric Van Cutsem

**Affiliations:** National Cancer Center Hospital East, Chiba, Japan; Department of Immunology, Nagoya University Graduate School of Medicine, Nagoya, Japan; University of Pisa, Pisa, Italy; City of Hope Comprehensive Cancer Center, Duarte, CA, USA; Medical College of Wisconsin, Milwaukee, WI, USA; Department of Haematology-Oncology, National University Cancer Institute, Singapore, National University Hospital, Singapore; Cancer and Stem Cell Biology Program, Duke-NUS Medical School, Singapore; Yong Loo Lin School of Medicine, National University of Singapore, Singapore; SmartAnalyst, an Ashfield Advisory Company, Gurugram, Haryana, India; University Hospitals Gasthuisberg Leuven and KU Leuven, Leuven, Belgium

**Keywords:** trifluridine, tipiracil, colorectal neoplasms, antineoplastic agents, antineoplastic drugs, review literature

## Abstract

We performed a systematic literature review to identify and summarize data from studies reporting clinical efficacy and safety outcomes for trifluridine/tipiracil (FTD/TPI) combined with other antineoplastic agents in advanced cancers, including metastatic colorectal cancer (mCRC). We conducted a systematic search on May 29, 2021, for studies reporting one or more efficacy or safety outcome with FTD/TPI-containing combinations. Our search yielded 1378 publications, with 38 records meeting selection criteria: 35 studies of FTD/TPI-containing combinations in mCRC (31 studies second line or later) and 3 studies in other tumor types. FTD/TPI plus bevacizumab was extensively studied, including 19 studies in chemorefractory mCRC. Median overall survival ranged 8.6-14.4 months and median progression-free survival 3.7-6.8 months with FTD/TPI plus bevacizumab in refractory mCRC. Based on one randomized and several retrospective studies, FTD/TPI plus bevacizumab was associated with improved outcomes compared with FTD/TPI monotherapy. FTD/TPI combinations with chemotherapy or other targeted agents were reported in small early-phase studies; preliminary data indicated higher antitumor activity for certain combinations. Overall, no safety concerns existed with FTD/TPI combinations; most common grade ≥ 3 adverse event was neutropenia, ranging 5%-100% across all studies. In studies comparing FTD/TPI combinations with monotherapy, grade ≥ 3 neutropenia appeared more frequently with combinations (29%-67%) vs. monotherapy (5%-41%). Discontinuation rates due to adverse events ranged 0%-11% for FTD/TPI plus bevacizumab and 0%-17% with other combinations. This systematic review supports feasibility and safety of FTD/TPI plus bevacizumab in refractory mCRC. Data on non-bevacizumab FTD/TPI combinations remain preliminary and need further validation.

## Teaser

The data reported here will inform treatment decision-making about the use of combination therapies that include trifluridine/tipiracil in the first-, second-, or third-line setting for the treatment gastrointestinal cancers across tumor types.

**Implications for Practice**

Trifluridine/tipiracil is approved as monotherapy in the treatment of metastatic colorectal and gastric cancers and has been explored in combinations with various antineoplastic combinations in clinical trials. This article provides an overview of the evidence for the activity and safety of these combinations across a variety of cancers. The data collected, summarized, and interpreted here will inform treatment of decision-making about the use of combination therapies that include trifluridine/tipiracil in the first-, second-, or third-line setting for the treatment gastrointestinal cancers across tumor types.

## Introduction

Fluoropyrimidines, including 5-fluorouracil and capecitabine, alone or as part of combination regimens, have formed the mainstay in treating gastrointestinal cancers.^1,2^ However, resistance to fluoropyrimidines remains a considerable barrier to effective treatment.^2^

Trifluridine/tipiracil (FTD/TPI or TAS-102; Taiho Oncology, Inc., Princeton, NJ, USA) is an oral cytotoxic agent comprising trifluridine, a thymidine analog, and tipiracil, a thymidine phosphorylase inhibitor.^3^ FTD/TPI has a unique mechanism of action distinguishing it from other fluoropyrimidines.^2^ FTD is incorporated into DNA, causing DNA dysfunction, and tipiracil increases the oral bioavailability of FTD.

In the phase III randomized RECOURSE trial, FTD/TPI monotherapy significantly improved survival versus placebo in patients with chemorefractory metastatic colorectal cancer (mCRC; after ≥ 2 prior systemic regimens). Median overall survival (OS) was 7.1 vs. 5.3 months for FTD/TPI versus placebo (hazard ratio [HR] 0.68 (95% confidence interval [CI], 0.58 − 0.81; *P* < .001). FTD/TPI also demonstrated a manageable safety profile, with hematologic and gastrointestinal-related adverse events (AEs) being the most common.^4^ In the phase III randomized TAGS trial, FTD/TPI monotherapy was associated with a significant survival benefit versus placebo (median OS, 5.7 vs. 3.6 months [95% CI, 4.8-6.2]; HR, 0.69 [95% CI, 0.56-0.85]; *P* < .001) in patients with metastatic gastric or gastroesophageal junction cancer (mGC/GEJC) whose disease progressed after ≥ 2 prior chemotherapy (chemo) regimens.^5^ As a result, FTD/TPI was approved as third- or later-line treatment for patients with mCRC (in 2015) and mGC/GEJC (in 2019).^6^

In addition to these two trials, multiple studies over recent years have evaluated the combination of FTD/TPI with targeted therapies, other chemotherapeutic agents, and immunotherapeutic agents, both in mCRC and other cancer types. The combination of FTD/TPI with the anti-vascular endothelial growth factor (VEGF) antibody bevacizumab (BEV) has shown promising results in patients with refractory mCRC in clinical trials,^7,8^ and FTD/TPI + BEV is now recommended in the National Comprehensive Cancer Network (NCCN) Clinical Practice Guidelines in Oncology (NCCN Guidelines) as a treatment option for patients with this disease.^9,10^

While there are systematic literature reviews (SLRs) and meta-analyses summarizing FTD/TPI monotherapy in CRC, ^11-13^ systematic reviews evaluating FTD/TPI-containing combination regimens across tumor types are rare. This SLR’s objective is to identify and summarize data from studies reporting clinical efficacy and safety outcomes for FTD/TPI in combination with other antineoplastic agents in various cancers, including CRC.

## Methods

Methods used in this unregistered SLR were prespecified and documented in a study protocol (supporting information). PRISMA reporting guidelines for systematic reviews^14,15^ and the Cochrane Handbook for Systematic Reviews of Interventions, version 5.1.0 guided reporting.^16^

### Literature Search Strategy

Studies of interest were randomized controlled trials (RCTs), nonrandomized clinical trials, and observational studies in abstract and full paper formats. The SLR was conducted on May 29, 2021, using search terms outlined in the supporting information, Supplementary Tables S1-S3. Data sources included MEDLINE (OvidSP); Embase (OvidSP); Cochrane Library (via Cochrane); conference proceedings (2018-2021) of the American Society of Clinical Oncology, European Society of Medical Oncology, and American Association for Cancer Research; the clinical trial registries: clinicaltrials.gov (https://clinicaltrials.gov/) and UMIN (https://www.umin.ac.jp/ctr/); bibliographies from relevant systematic reviews; grey literature sources; and clinical guidelines.

Following Cochrane guidelines, Population, Intervention, Comparator, Outcome, and Study Design (PICOS) methodology were used to build the search strategies.

### Screening and Study Selection

**Figure 1.**
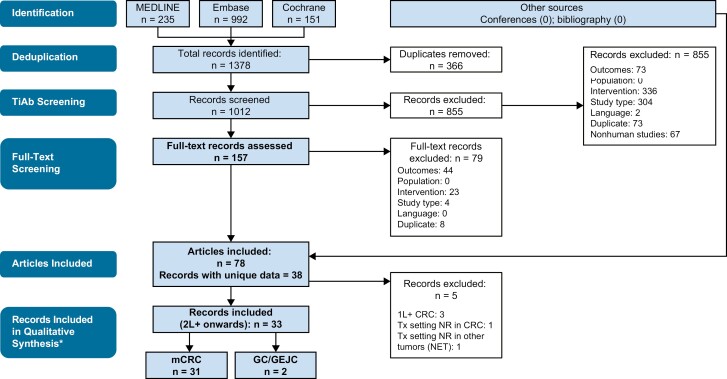
Selection process for studies included in the systematic literature review (PRISMA diagram). *Studies in second-line or later settings were included for qualitative synthesis. Studies based on frontline treatment or where line of treatment was not explicitly reported were summarized separately. Abbreviations: 1L, first line; 2L, second line; GC, gastric cancer; GEJC, gastro-oesophageal junction carcinoma; mCRC, metastatic colorectal cancer; NET, neuroendocrine tumors; NR, not reported; TiAb, title/abstract; Tx, treatment.

Inclusion and exclusion criteria developed using the PICOS approach were applied to shortlist publications of interest for studies reporting clinical efficacy, safety, and health-related quality of life (HRQoL) outcomes for FTD/TPI combined with other antineoplastic agents to treat patients with cancer (Supplementary Table S4). Using these criteria, 2 researchers independently screened abstracts and then full-text articles in a 2-stage process, with a third reviewer adjudicating any differences. Only studies that fulfilled the inclusion and exclusion criteria and reported one or more defined outcomes were included in the analysis. Identification of records is shown (Fig. 1), aligned with PRISMA statement recommendations.^14^

### Qualitative Synthesis of Data

Findings were tabulated and summarized. One reviewer extracted data from the included records into Microsoft Excel tables, which were then checked and validated by a second reviewer. Outputs included a trial design overview, patient population (tumor type and stage), sample size, follow-up duration, intervention, comparator, primary and secondary endpoints including but not limited to clinical efficacy, safety, and HRQoL. Results were collated and studies were characterized by cancer type, line of treatment, and finally by the type of FTD/TPI combination partner. As this analysis was designed to provide a qualitative review, median values and ranges were provided for survival outcomes.

The study followed a prespecified protocol as a qualitative rather than quantitative analysis. A quantitative meta-analysis was not conducted due to considerable differences between the studies in terms of study design, disease, intervention, and line of treatment. For various combinations of disease, interventions, and lines of treatment, there was an insufficient number of studies for all the groups other than for the FTD/TPI and BEV combination in 2L+ or 3L+ mCRC. Patient characteristics in these individual studies were sporadically reported, and for those that were reported, too diverse to be appropriately combined in a meta-analysis. The outcomes being reported were summarized as median time-to-event endpoints (OS/PFS), which often follow skewed distributions and assumptions valid in meta-analyses of means that may not have been appropriate for medians. For these reasons, a qualitative synthesis of the data was performed.

### Risk of Bias Quality Assessment

Criteria from the Cochrane risk-of-bias tool for RCTs^16,17^ and the Downs and Black checklist for non-RCTs^17^ were used to assess the risk of bias in RCTs and non-RCTs, respectively. In addition, the Newcastle-Ottawa Scale was used to evaluate single-arm observational studies.^18^ Two reviewers assessed risk of bias, with disagreement resolved by discussion.

## Results

### Study Selection

In total, 1378 records were identified across all databases searched (Fig. 1). Of these, 366 were duplicates between sources, and another 934 records were excluded primarily because of interventions or study types not of interest. After screening both abstracts and full-text articles for eligibility, 78 articles were included and yielded 38 records with unique data.

Overall, 31 records included studies of FTD/TPI-containing combinations used in second- or third-line or later (2L+ or 3L+) settings in mCRC, ^7,8,19-47^ 4 records detailed studies of FTD/TPI combinations in the first-line (1L) setting in mCRC (*n* = 3) ^48-50^ or where treatment setting was not reported in mCRC (*n* = 1),^51^ 2 were studies of FTD/TPI-containing combinations used in the 2L + setting in mGC/GEJC,^52,53^ and one study evaluated FTD/TPI plus temozolamide (TEM) in patients with advanced neuroendocrine tumors (previous treatment unspecified).^54^

Data in mCRC, and particularly those in previously treated mCRC, were summarized separately for this analysis, as this constituted the largest subset of the results.

### FTD/TPI-Containing Combinations in 2L+ or 3L+ mCRC

The designs and the key characteristics of the 31 studies evaluating the use of FTD/TPI combination therapies in 2L+ or 3L+ mCRC identified in the review are presented in Table 1. Ten studies (1 RCT, 2 non-RCTs, and 7 retrospective studies) analyzed FTD/TPI + BEV in 2L+ mCRC, ^7,8,20,22-24,27,33,36,46^ 9 studies (5 non-RCTs and 4 retrospective studies) analyzed FTD/TPI + BEV in 3L+ mCRC,^25,28,29,31,34,37,38,43,47^ and 4 studies analyzed FTD/TPI in combination with chemotherapeutic agents such as irinotecan (IRI) and oxaliplatin (OXA) (2 in 2L+ and 2 in 3L+).^19,26,39,42^ Three studies analyzed FTD/TPI + BEV + chemo (OXA or IRI) in mCRC, one reported data from FTD/TPI + nivolumab (NIVO) + OXA^32,40,41^; and 5 studies (non-RCTs) evaluated FTD/TPI in combination with targeted therapies, such as panitumumab (PAN), nintedanib (NIN), regorafenib (REG), and murlentamab, or immunotherapeutic agents, including NIVO.^21,30,35,44,45^ One study whose treatment setting was not specified, and therefore not included among these 31 studies, evaluated FTD/TPI with or without ramucirumab in advanced mCRC.^51^

**Table 1.** Studies of FTD/TPI-containing combination regimens in metastatic colorectal cancer.

Study	Study type/phase	Population specifics	Intervention 1, *n*	Intervention 2, *n*	Treatment setting	Reported study endpoints^a^	Study location	Median follow-up, mo
*FTD/TPI + BEV*								
Van Cutsem et al^48,50,55^	Phase II RCT	mCRC	FTD/TPI + BEV *n* = 77	CAP + BEV *n* = 76	1L	PE: PFS SE: OS, QoL, safety	12 countries^b^	NA
Oki et al^49^	Phase II non-RCT	mCRC ≥70 years	FTD/TPI + BEV *n* = 39	—	1L	PE: PFS SE: OS, ORR, safety	Japan	18.9
Pfeiffer et al^8^	Phase II RCT	mCRC	FTD/TPI + BEV *n *= 46	FTD/TPI mono *n* = 47	2L+	PE: PFS SE: OS, ORR, DCR, safety	Denmark	10·0
Kuboki et al^7^	Phase I/II non-RCT	mCRC	FTD/TPI + BEV *n* = 25	—	2L+	PE: PFS at 16 wks SE: PFS, ORR, DCR, TTF, OS, PK, AEs	Japan	11·4
Takahashi et al^46^	Phase II non-RCT	mCRC	FTD/TPI + BEV *n* = 97 (safety data set, *n* = 102)	—	2L+	PE: DCR by RAS status SE: DCR overall, PFS, OS, ORR overall and by RAS status	Japan	15.8
Nose et al^36^	Retro Obs	mCRC	FTD/TPI + BEV *n* = 32	FTD/TPI mono *n* = 24	2L+	PE: PFS SE: OS, safety	Japan	11.6; 6.3
Fujii et al^33^	Retro Obs	mCRC	FTD/TPI + BEV *n* = 21	FTD/TPI mono *n* = 36	2L+	PE: OS SE: ORR, TTF	Japan	14.8
Hisamatsu et al^27^	Retro Obs	mCRC	FTD/TPI + BEV *n* = 24	—	2L+	PE: PFS SE: ORR, DCR, OS, safety	Japan	NA
Ishikawa et al^22^	Retro Obs	mCRC	FTD/TPI + BEV *n *= 22	FTD/TPI mono *n* = 23	2L+	DCR, PFS, OS, AEs	NA	NA
Makiyama et al^23^	Retro Obs	mCRC	FTD/TPI + BEV *n* = 11	FTD/TPI mono *n* = 33	2L+	PFS, OS, safety	Japan	NA
Yasuda et al^24^	Retro Obs	mCRC	FTD/TPI + BEV *n* = 33	—	2L+	Safety	Japan	NA
Ota et al^20^	Retro Obs	mCRC	FTD/TPI ± BEV *n* = 14	—	2L+	PFS, OS, safety	Japan	10.3
Miano et al^34^	Non-RCT	mCRC	FTD/TPI + BEV *n* = 15	—	3L+	PE: PFS SE: RR, OS, grade 3 neutropenia	Italy	100
Satake et al^37^	Phase Ib/II non-RCT	mCRC	FTD/TPI + BEV *n* = 44	—	3L+	PE: PFS at 16 wks SE: OS, PFS, TTF, ORR, DCR, safety	Japan	15.36
Yoshida et al^31^	Phase II non-RCT	mCRC	FTD/TPI + BEV *n* = 45	—	3L+	PE: PFS SE: RR, DCR, OS, safety	Japan	NA
Ishizaki et al^43^	Phase II non-RCT	mCRC	FTD/TPI + BEV *n* = 19	—	3L+	PE: PFS SE: OS, ORR, DCR, AEs, time to ECOG PS ≥ 2	Japan	11.5
Yoshida et al^47^	Phase II non-RCT	mCRC	FTD/TPI + BEV *n* = 32	—	3L+	PE: PFS SE: TTF, RR, OS, AEs	Japan	NA
Shibutani et al^38^	Retro Obs	mCRC	FTD/TPI + BEV *n* = 36	FTD/TPI mono *n* = 26	3L+	ORR, PFS, OS, safety	Japan	NA
Matsuhashi et al^29^	Retro Obs	mCRC	FTD/TPI + BEV *n* = 17	—	3L+	ORR, DCR, PFS, OS, safety	Japan	NA
Kotani et al^28^	Retro Obs	mCRC	FTD/TPI + BEV *n* = 60	FTD/TPI mono *n* = 66	3L+	PFS, ORR, DCR, OS, AEs	Japan	7.1; 7.2
Yoshida et al^25^	Retro Obs	mCRC	FTD/TPI + BEV *n* = 25	FTD/TPI mono *n* = 16	3L+	ORR, PFS, OS, AEs	Japan	NA
*FTD/TPI + BEV + Chemo*								
Varghese et al^40^	Phase I non-RCT	mCRC	FTD/TPI + BEV + IRI *n* = 24	FTD/TPI + IRI *n* = 26	2L+	PE: Safety, MTD SE: Safety	NR	NA
Yamazaki et al^41^	Phase II non-RCT	mCRC	FTD/TPI + BEV + IRI *n* = 18	—	2L+	PE: ORR SE: Safety	Japan	NA
Bordonaro et al^32^	Phase I non-RCT	mCRC	FTD/TPI + BEV + OXA *n* = 37	FTD/TPI + OXA + NIVO *n* = 17	3L+	ORR, DCR, PFS, OS, safety	France, Spain, Italy, Germany, Austria, Hungary, UK	NA
*FTD/TPI + Chemo*								
Doi et al^19^	Phase I non-RCT	mCRC	FTD/TPI + IRI *n* = 10	—	2L+	PE: RD, safety SE: Efficacy, PK	Japan	33.7
Argilés et al^26^	Phase I non-RCT	mCRC	FTD/TPI + OXA *n* = 24	—	2L+	PE: MTD, RD, safety SE: PK, anti-tumor activity	France, Spain	NA
Suenaga et al^39^	Phase I non-RCT	mCRC	FTD/TPI + OXA *n* = 12	—	3L+	Response, PFS, OS, safety	Japan	13.8
Cecchini et al^42^	Phase Ib/II non-RCT	mCRC	FTD/TPI + OXA *n* = 41	—	3L+	PE: ORR SE: PFS, OS, DCR, DOR, safety	NR	6.8
*FTD/TPI + targeted therapy*								
Kato et al^44^	Phase I/II non-RCT	mCRC	FTD/TPI + PAN *n* = 54 (safety population: n = 55)	—	2L+	PE: PFS at 6 mo SE: PFS, OS, ORR, DCR, TTF, safety	Japan	16.5
Van Cutsem et al^30^	Phase II non-RCT	mCRC	FTD/TPI + MUR *n* = 15	MUR *n* = 14	2L+	Response, PFS, OS, PD, safety	Belgium, Czech Rep.	NA
Yamazaki et al^21^	Phase I/II non-RCT	mCRC	FTD/TPI + NIN *n* = 52	—	2L+	PE: PFS at 16 wks SE: OS, DCR, ORR, safety	Japan	NA
Moehler et al^35^	Phase I non-RCT	mCRC	FTD/TPI + REG *n* = 12	—	3L+	PE: MTD SE: DCR, PFS, OS, safety	Germany	NA
Patel et al^45^	Phase II non-RCT	mCRC	FTD/TPI + NIVO *n* = 18	—	3L+	PE: irORR SE: ORR, PFS, DCR, OS, safety	USA	NA
Kasper et al^51^	Phase IIb RCT	mCRC	FTD/TPI + RAM *n* = 40	FTD/TPI *n* = 40	NR	PE: OS SE: ORR, DCR, PFS, safety	Germany	NA

^a^Where PE and SE are not specified in the column, study endpoints were not classified as primary or secondary.

^b^Australia, Belgium, Brazil, Denmark, France, Germany, Italy, The Netherlands, Poland, Russia, Spain, and UK.

Abbreviations: 1L, first line; 2L, second line; 3L, third line; AE, adverse event; BEV, bevacizumab; CAP, capecitabine; DCR, disease control rate; DOR, duration of response; ECOG PS, Eastern Cooperative Oncology Group performance status; FTD/TPI, trifluridine/tipiracil; IRI, irinotecan; irORR, immune-related objective response rate; mCRC, metastatic colorectal cancer; mo, months; Mono, monotherapy; MTD, maximum tolerated dose; MUR, murlentamab; NA, not available; NIN, nintedanib; NIVO, nivolumab; Non-RCT, nonrandomized controlled trial; NR, not reported; ORR, overall response rate; OS, overall survival; OXA, oxaliplatin; PAN, panitumumab; PD, pharmacodynamics; PE, primary endpoint; PFS, progression-free survival; PK, pharmacokinetics; QoL, quality of life; RCT, randomized controlled trial; RD, recommended dose; REG, regorafenib; Retro Obs, retrospective observational, RR, response rate; SE, secondary endpoint(s); TTF, time to treatment failure; wks, weeks.

Among the 31 studies, 26 reported one or more efficacy outcomes. Most studies (21/31) were conducted in Japan; all clinical trials were phase I or II, with patient population sizes ranging from 10 to 97 patients. While several retrospective studies (*n* = 7) evaluated FTD/TPI monotherapy concurrently with FTD/TPI-containing combination regimens, only one RCT^8^ was powered for statistical comparison of outcomes with FTD/TPI + BEV versus those with FTD/TPI monotherapy.

### Efficacy in Patients With Previously Treated mCRC

**Figure 2.**
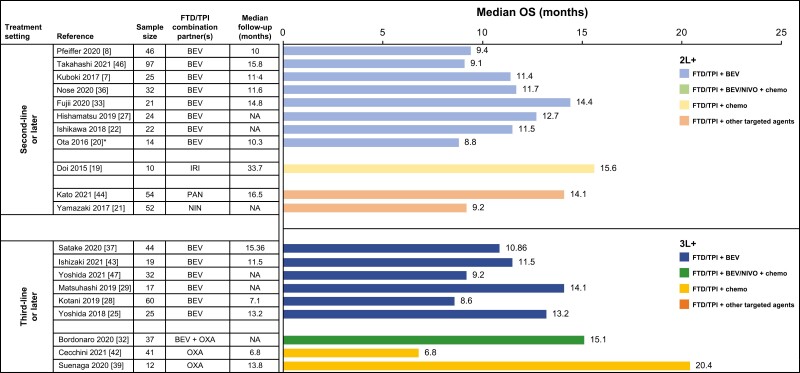
Median OS with FTD/TPI-containing combination regimens in patients with metastatic colorectal cancer in second- or third-line settings. *Data pertains to patients who received FTD/TPI ± BEV. Abbreviations: 2L, second line; 3L, third line; BEV, bevacizumab; chemo, chemotherapy; FTD/TPI, trifluridine/tipiracil; IRI, irinotecan; NA, not applicable; NIN, nintedanib; NIVO, nivolumab; OS, overall survival; OXA, oxaliplatin; PAN, panitumumab.

**Figure 3.**
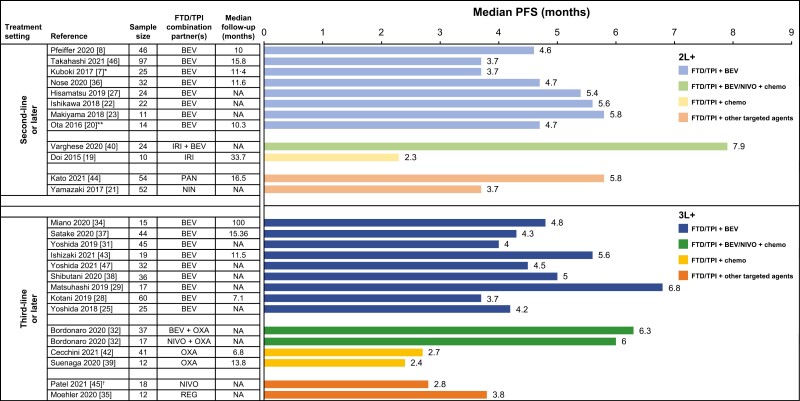
Median PFS with FTD/TPI-containing combination regimens in patients with metastatic colorectal cancer in second- or third-line settings. *Median PFS was 3.7 by central assessment and 5.6 months by investigator assessment. **Data pertain to patients receiving FTD/TPI ± BEV. ^†^Median PFS was 2.2 months per immune-related response criteria and 2.8 months per response evaluation criteria in solid tumors (RECIST). Abbreviations: 2L, second line; 3L, third line; BEV, bevacizumab; chemo, chemotherapy; FTD/TPI, trifluridine/tipiracil; IRI, irinotecan; NA, not applicable; NIN, nintedanib; NIVO, nivolumab; OXA, oxaliplatin; PAN, panitumumab; PFS, progression-free survival; REG, regorafenib.

Among patients with mCRC treated with FTD/TPI + BEV in the 2L+ setting, median OS ranged from 8.8 to 14.4 months (Fig. 2; Supplementary Table S5), and median progression-free survival (PFS) ranged from 3.7 to 5.8 months (Fig. 3, Supplementary Table S6).^7,8,20,22,23,27,33,36,46^ In one RCT, FTD/TPI + BEV treatment was associated with significantly longer OS (HR, 0.55, 95% CI, 0.32-0.94) and PFS (HR, 0.45, 95% CI, 0.29-0.72) compared with FTD/TPI monotherapy.^8^ In retrospective observational studies in the 2L+ setting, OS HRs for FTD/TPI + BEV versus FTD/TPI monotherapy ranged from 0.24 to 0.30,^23,33,36^ and PFS HRs ranged from 0.28 to 0.34.

Ranges of OS and PFS in patients with mCRC treated with FTD/TPI + BEV in the 3L+ setting were similar to that observed in the 2L+ setting (median OS, 8.6 − 14.1 months; median PFS, 3.7 − 6.8 months; Figs. 2 and 3; Supplementary Tables S5 and S6).^25,28,29,31,34,37,43,47^ A single retrospective observational study showed PFS benefit with FTD/TPI + BEV treatment compared with FTD/TPI monotherapy (HR 0.69) in patients with mCRC treated in the 3L+ setting.^28^ Together, these data indicated a trend toward OS and PFS benefit with FTD/TPI + BEV compared with FTD/TPI monotherapy in patients with previously treated mCRC. Response rates and disease control rates (DCRs) followed a similar trend, although objective response rates (ORRs) were low overall in patients with previously treated mCRC. In 2L+ mCRC, ORRs with FTD/TPI + BEV ranged from 0% to 3% (Supplementary Fig. S1A) and DCRs ranged from 61% to 76% (Supplementary Fig. S2A).^7,8,22,27,33,46^ In the 3L+ setting, ORRs ranged from 0% to 8%^25,28,29,31,37,38,47^ (except for a small study, which reported an ORR of 26%^43^) (Supplementary Fig. S1A), and DCRs ranged from 53% to 73% (Supplementary Fig. S2A).

The triplet combination of BEV + FTD/TPI + another chemotherapeutic agent was evaluated in three small studies, with two reporting efficacy outcomes. A median OS of 15.1 months and median PFS of 6.3 months were noted with FTD/TPI + BEV + OXA in 3L+ mCRC (Figs. 2 and 3; Supplementary Tables S7 and S8),^32^ and a median PFS of 7.9 months was noted with FTD/TPI + BEV + IRI in 2L+ mCRC.^40^ DCRs ranged from 83% to 89% among patients treated with FTD/TPI + BEV + chemo in the 2L+ and 3L+ settings (Supplementary Fig. S2B).

Among FTD/TPI-containing combinations with other targeted therapies, 2 studies (APOLLON evaluating FTD/TPI + PAN and N-task force evaluating FTD/TPI + NIN) enrolled ≈ 50 patients. FTD/TPI + PAN resulted in a median OS of 14.1 months and a median PFS of 5.8 months, whereas FTD/TPI + NIN resulted in a median OS of 9.2 months and median PFS of 3.7 months (Figs. 2 and 3; Supplementary Tables S7 and S8). FTD/TPI + PAN and FTD/TPI + NIN were associated with ORRs of 37% and 8% and DCRs of 82% and 69%, respectively (Supplementary Figs. S1B and S2B).^21,44^ A small study (REMETY; *n* = 12), which evaluated FTD/TPI + REG, reported a median PFS of 3.8 months.^35^

Three of 4 studies evaluating the combination of FTD/TPI with chemotherapeutic agents reported survival data.^19,26,39,42^ In a small study of FTD/TPI + IRI (*n* = 10 [9 evaluable patients]) in 2L+ mCRC, median PFS was 2.3 months and median OS was 15.6 months (Figs. 2 and 3; Supplementary Tables S7 and S8).^19^ In 2 studies of FTD/TPI + OXA, median OS was 6.8 months (*n* = 41) and 20.4 months (*n* = 12); median PFS was 2.7 and 2.4 months, respectively.^39,42^ DCRs ranged from 56% to 68% among patients treated with FTD/TPI + chemo (Supplementary Fig. S2B).

The combination of FTD/TPI + NIVO + OXA was associated with a median PFS of six months, and an ORR of 7%, whereas FTD/TPI + NIVO was associated with a median PFS of 2.8 months and a 0% ORR (Figs. 2 and 3; Supplementary Tables S7 and S8).^32,45^

### Safety in Patients With Previously Treated mCRC

Among patients treated with FTD/TPI + BEV combinations in both 2L+ and 3L+ settings, neutropenia was the most frequently observed grade ≥ 3 AE (Table 2). ^7,8,28,29,36-38,43,46,47^ In the phase II RCT comparing FTD/TPI + BEV with FTD/TPI monotherapy, the incidence of grade 3 or 4 neutropenia was higher with FTD/TPI + BEV (67%) than with FTD/TPI alone (38%).^8^ However, in this study, the overall rates of serious AEs were similar in the FTD/TPI and FTD/TPI + BEV groups, and similar numbers of patients discontinued because of AEs. Across retrospective observational studies that evaluated both FTD/TPI and FTD/TPI + BEV in patients with previously treated mCRC,^22,28,29,33,36,38^ rates of grade 3 or higher neutropenia ranged from 5% to 41% in the FTD/TPI group and 29% to 53% in the FTD/TPI + BEV group. Other key AEs experienced by patients receiving FTD/TPI + BEV included fatigue, nausea, and diarrhea; however, incidences of grade ≥ 3 events of these AEs remained low (Table 2). The proportions of patients discontinuing treatment due to an AE were low, ranging from 0% to 11% across various studies.

**Table 2.** Summary of safety in patients with metastatic colorectal cancer treated with FTD/TPI plus bevacizumab in second- or third-line settings.

Study	Study design	Tx setting	Regimen	Sample size	Grade ≥ 3 AEs, %								% disc
					Neutropenia	FN	Diarrhea	Constipation	Nausea	Vomiting	Asthenia	Fatigue	
Pfeiffer et al ^8^	RCT	2L+	FTD/TPI + BEV	46	67	6	9	NA	2	4	NA	7	2
Takahashi et al^46^	Non-RCT	2L+	FTD/TPI + BEV	102	54	4	5	NA	5	NA	NA	1	9
Kuboki et al^7^	Non-RCT	2L+	FTD/TPI + BEV	25	72	16	0	NA	0	0	NA	0	0
Nose^36^	Ret Obs	2L+	FTD/TPI + BEV	32	53	NA	0	NA	0	NA	NA	6	0
Satake^37^	Non-RCT	3L+	FTD/TPI + BEV	44	16	0	5	NA	7	0	NA	0	11
Ishizaki^43^	Non-RCT	3L+	FTD/TPI + BEV	19	5	NA	0	NA	0	0	NA	NA	5
Yoshida^47^	Non-RCT	3L+	FTD/TPI + BEV	32	47	NA	0	NA	6	0	NA	3	3
Shibutani^38^	Ret Obs	3L+	FTD/TPI + BEV	36	39	0	0	NA	0	0	NA	3	NA
Matsuhashi^28^	Ret Obs	3L+	FTD/TPI + BEV	17	41	0	0	NA	0	5	NA	0	NA
Kotani^27^	Ret Obs	3L+	FTD/TPI + BEV	60	50	3	0	NA	0	0	NA	0	NA

Abbreviations: 2L, second line; 3L, third line; AE, adverse event; BEV, bevacizumab; disc, AE-related discontinuation; FN, febrile neutropenia; FTD/TPI, trifluridine/tipiracil; NA, not available; Non-RCT, nonrandomized controlled trial; RCT, randomized controlled trial; Ret Ob, retrospective observational study; Tx, treatment.

Among patients treated with other FTD/TPI combinations in both 2L+ and 3L+ settings, neutropenia was again the most frequent grade ≥ 3 AE observed (incidence ranging from 17% to 100%), and the proportions of patients discontinuing treatment due to an AE ranged from 0% to 17% (Table 3).^19,26,40,42,45^ Grade ≥ 3 neutropenia was reported at an incidence of 100% with the combination of FTD/TPI with IRI at the highest dose level evaluated in a dose-escalation study.^19^

**Table 3.** Summary of safety in patients with metastatic colorectal cancer treated with other FTD/TPI combinations in second- or third-line settings.

Study	Study design	Tx setting	Regimen	Sample size	Follow-up (median months)	Grade ≥ 3 AEs, %								% disc
						Neutropenia	FN	Diarrhea	Constipation	Nausea	Vomiting	Asthenia	Fatigue	
*FTD/TPI + chemo + BEV*														
Varghese^40^	Non-RCT	2L+	FTD/TPI + IRI + BEV	24	NR	42	NR	12	0	4	8	NA	8	0
*FTD/TPI + chemo*														
Doi^19^	Non-RCT	2L+	FTD/TPI + IRI	10	33.7	100	30	0	0	0	0	NA	NA	0
Varghese^40^	Non-RCT	2L+	FTD/TPI + IRI	26	NA	23	NA	0	0	12	12	NA	15	4
Argilés^26^	Non-RCT	2L+	FTD/TPI + OXA	24	NA	17	NA	0	NA	0	4	4	NA	17
Cecchini^42^	Non-RCT	3L+	FTD/TPI + OXA	41	6.8	20	NA	2	NA	0	0	NA	2	7
Suenaga^39^	Non-RCT	3L+	FTD/TPI + OXA	12	13.8	25	0	0	0	8	0	NA	0	NA
*FTD/TPI + targeted/immunotherapy*														
Kato^44^	Non-RCT	2L+	FTD/TPI + PAN	55	16.5	47	11	2	NA	2	2	NA	4	4
Patel^45^	Non-RCT	3L+	FTD/TPI + NIVO	18	NA	28	NA	17	NA	11	6	6	11	0

Abbreviations: 2L, second line; 3L, third line; AE, adverse event; BEV, bevacizumab; chemo, chemotherapy; disc, discontinuation; FN, febrile neutropenia; FTD/TPI, trifluridine/tipiracil; IRI, irinotecan; mCRC, metastatic colorectal cancer; NA, not available; NIVO, nivolumab; Non-RCT, nonrandomized controlled trial; OXA, oxaliplatin; PAN, panitumumab; Tx, treatment.

The standard recommended dose of FTD/TPI requires either no or slight adjustment when combined with other antineoplastic agents. A dose escalation study indicated that FTD/TPI is safe in treating mCRC at the recommended dose of 35 mg/m² bid in combination with 85 mg/m² of OXA Q2W,^26^ while other studies indicated a lower maximum tolerated dose of FTD/TPI (25 mg/m² bid) when combined with 180 mg/m² IRI Q2W^40^ or with 120 mg REG daily.^35^

### FTD/TPI + BEV in 1L mCRC

Two studies evaluated FTD/TPI + BEV in the 1L setting in mCRC (Table 1). In the noncomparative phase II RCT TASCO1, in patients with mCRC ineligible for full-dose combination chemotherapy with irinotecan or oxaliplatin or for curative resection of metastatic lesions, FTD/TPI + BEV (*n* = 77) and capecitabine + BEV (*n* = 76) were respectively associated with a median PFS of 9.2 and 7.8 months, median OS of 18 and 16.2 months, ORRs of 34% and 30%, and DCRs of 86% and 78%. In the FTD/TPI + BEV and capecitabine + BEV groups, most frequent grade ≥ 3 AEs, were neutropenia (47% and 5%), hand-foot syndrome (0% and 12%) and diarrhea (0% and 8%).^48^ In extended follow-up, median OS was 22.3 months with FTD/TPI + BEV and 17.7 months with capecitabine + BEV.^50,55^ Overall, these data indicated clinical activity of the FTD/TPI + BEV regimen in untreated mCRC, with efficacy similar to that of capecitabine + BEV. In a smaller phase II trial in patients with mCRC aged ≥ 70 years, including those ineligible or eligible for (but opted not to receive) oxaliplatin- or irinotecan-containing regimens (*n* = 37), FTD/TPI + BEV as IL treatment resulted in a median OS of 22.4 months, median PFS of 9.4 months, an ORR of 41%, and a DCR of 87%. Most (72%) patients experienced grade ≥ 3 neutropenia.^49^

### FTD/TPI-Containing Combinations in Other Tumor Types

Two studies evaluated FTD/TPI-containing combination regimens in mGC/GEJC in the 2L+ setting. A phase II clinical trial that evaluated FTD/TPI + ramucirumab in patients with mGC/GEJC who were previously treated (*n* = 64) resulted in an ORR of 13% and a DCR of 81%. In total, 78% of patients experienced grade ≥ 3 neutropenia. The efficacy and safety data were consistent regardless of previous ramucirumab exposure, and the authors concluded that this regimen had clinical activity in this population.^53^ Preliminary results from a phase I/II clinical trial assessing FTD/TPI + IRI in patients with previously treated GC (*n* = 11) indicated a median PFS of 3 months, and a median OS of 10.2 months. Overall, 91% of patients had grade ≥ 3 neutropenia.^52^

Separately, one phase I dose-escalation trial evaluated FTD/TPI + TEM in patients with advanced neuroendocrine tumors (*n* = 15; prior treatment unspecified). In this trial, an ORR of 8% and a DCR of 92% was observed; 33% experienced grade ≥ 3 neutropenia.^54^

## Discussion

To our knowledge, this SLR is the first to summarize all published studies of FTD/TPI-containing combination regimens across tumors. Most studies evaluating FTD/TPI-containing combination regimens were conducted in patients with chemorefractory mCRC, with FTD/TPI + BEV being the most extensively studied. This SLR suggests that adding BEV to FTD/TPI yielded a clinically meaningful benefit for disease control ^7,8,22,25,27-29,31,33,37,38,43,46,47^ and improved survival outcomes versus FTD/TPI alone^9^ in patients with chemorefractory mCRC. Median PFS was approximately 4 to 6 months, and median OS was around 8 to 14 months with FTD/TPI + BEV. ^7,8,20,22,23,25,27-29,33,34,36-38,43,46,47^ A recent press release announced positive outcomes in the Phase III randomized SUNLIGHT trial (NCT04737187), which evaluated FTD/TPI + BEV versus FTD/TPI monotherapy in refractory mCRC, although data remain pending (Table 4). These data are in line with recent NCCN Guidelines^®^ of FTD/TPI with or without BEV for patients with chemorefractory mCRC.^9,10^

**Table 4.** Ongoing and recently reported trials of FTD/TPI in combination with other agents in mCRC and other advanced solid tumors (up to date July 2022).

Study name	Study ID	Setting	Agent(s)	Description and location(s)	Study status
*Advanced mCRC*					
SOLSTICE^56^	NCT03869892 EudraCT: 2017-004059-22	1L mCRC	FTD/TPI + BEV versus capecitabine + BEV	Randomized phase III (non-US international)	Active, not recruiting; preliminary results^56^
TOBACO	NCT05077839	1L mCRC	FTD/TPI + oxaliplatin and BEV versus XELOX + BEV	Parallel, randomized, standard-control phase II study (China)	Recruiting
TriComB	NCT04564898 EudraCT: 2020-000923-37	1L mCRC	FTD/TPI + Capecitabine and BEV	Single-arm, phase I/II (Italy)	Recruiting
FIRE-8	NCT05007132 EudraCT: 2019-004223-20	1L mCRC	FTD/TPI + panitumumab versus FTD/TPI + BEV	Randomized, open label, multicenter phase II (Germany)	Recruiting
TASCO1	NCT02743221	1L mCRC	FTD/TPI + BEV versus capecitabine + BEV	Open-label, randomized phase II	Completed
SUNLIGHT	NCT04737187 EudraCT: 2020-001976-14	1L, 2L, or 3L mCRC (refractory mCRC)	FTD/TPI + BEV versus FTD/TPI	Randomized phase III (US/global)	Active, not recruiting
(N/A)	UMIN000041621	mCRC (all lines)	FTD/TPI + BEV	Pooled analysis of 5 trials (Japan)	Preinitiation
3T Study	NCT05356897	2L+ mCRC	FTD/TPI + tucatinib + trastuzumab	Single-arm phase II study (US)	Not yet recruiting
(N/A)	NCT04294264	2L+ mCRC	FTD/TPI + oxaliplatin	Single-arm phase II study (US)	Recruiting
(N/A)	NCT02848443	2L+ mCRC	FTD/TPI + oxaliplatin (+/− BEV or nivolumab)	Phase I (UK/Europe)	Completed
TABAsCO	NCT04109924	2L+ mCRC	FTD/TPI + BEV + IRI	Single-arm phase II study (US)	Recruiting
WJOG14520G	UMIN000044136	2L+ mCRC	FTD/TPI + BEV	Retrospective study (Japan)	No longer recruiting
HS-CA102N-101	NCT03616574	2L+ locally advanced/metastatic CRC	FTD/TPI + CA102N	Phase I/II	Enrolling by invitation
(N/A)	NCT04511039	2L+ locally advanced/mCRC or GEJC	FTD/TPI + Talazoparib	Phase I (US)	Recruiting
TASKIN	NCT05201352	2L+ mCRC	FTD/TPI + XB2001 versus FTD/TPI + placebo	Randomized (1:1 ratio), double-blind, noncomparative, multi-centre phase II study (France)	Not yet recruiting
(N/A)	NCT03317119	2L+ mCRC (unresectable)	FTD/TPI + trametinib	Phase I study (US)	Active, not recruiting
(N/A)	NCT05130060	3L+ mCRC	PolyPEPI1018 vaccine + FTD/TPI	Phase I study (US)	Recruiting
COLSTAR	NCT05223673 EudraCT: 2021-003151-41	3L+ *KRAS/NRAS* and *BRAF* wt mCRC	Futuximab/modotuximab + FTD/TPI versus FTD/TPI	Randomized, open-label, multicenter, 2-arm phase III safety lead-in study	Recruiting
RM-110	NCT04073615	3L+ mCRC	Rivoceranib + FTD/TPI versus monotherapies)	Phase I/II multicenter, open-label, randomized study (US)	Active, not recruiting
VELO	NCT05468892 EudraCT: 2018-001600-12	3L+ mCRC (major response to prior 1L; progression on 2L)	FTD/TPI + panitumumab versus FTD/TPI	Open label, phase II randomized study	Completed
(N/A)	NCT04868773	3L+ mCRC	FTD/TPI + cabozantinib	Phase I study (US)	Recruiting
TACTIC	NCT05266820	3L+ mCRC	FTD/TPI + thalidomide versus FTD/TPI	Phase II study (China)	Recruiting
CT001	NCT05155124	3L mCRC	FTD/TPI + cetuximab	Phase I study (China)	Recruiting
*Other solid tumors*					
ONC001	NCT04393298	1L+ advanced solid tumors (including mCRC, mGC/mGEJC, others)	UCB6114 ± FTD/TPI	Phase I/II nonrandomized, open-label study (US/UK) (US/UK)	Recruiting
(N/A)	NCT04808791	1L locally advanced/metastatic GC/GEJ adenocarcinoma	IRI + FTD/TPI + oxaliplatin	Single-arm, phase II study (Canada)	Not yet recruiting
(N/A)	NCT04097028	1L resectable esophageal/GEJ adenocarcinoma	FTD/TPI + Oxaliplatin	Phase II trial (US)	Recruiting
MC1941	NCT04072445	2L+ advanced refractory biliary tract cancer	FTD/TPI + IRI	Single-arm phase II study (US)	Active, not recruiting
(N/A)	NCT03368963	2L+ advanced GI cancers (dose expansion phase only: pancreatic/CRC)	TAS102 + nanoliposomal IRI	Phases I/II trial (US)	Recruiting
ACCRU-GI-1810	NCT04660760	2L+ advanced GC/GEJC	FTD/TPI + ramucirumab versus paclitaxel + ramucirumab	Phase II randomized trial (US)	Recruiting
LonGas^57^	EudraCT: 2018-004845-18	Platinum-refractory GEJ adenocarcinoma	FTD/TPI ± BEV	Randomized phase III study (Denmark)	Completed; preliminary results^57^
RE-ExPEL	EudraCT: 2020-001075-32	Advanced/metastatic GC/GEJC	Ramucirumab beyond progression plus TAS-102	Pilot study (Germany)	Ongoing
ACOTAS_G098	EudraCT: 2020-004636-25	mCRC/mGC/mGEJC	FTD/TPI ± oxaliplatin	Phase II cardiovascular safety study (France)	Ongoing
ACE1100-01	EudraCT: 2021-003799-15	3L advanced GC	FTD/TPI + ASC-201 versus FTD/TPI	Randomized, double-blind phase II study (Spain)	Ongoing
TRITICC	NCT04059562	2L cholangiocarcinoma	FTD/TPI + IRI	Prospective, single arm, open label, exploratory, multi-centre pilot study (Germany)	Recruiting
(N/A)	EudraCT: 2018-002936-26	2L+ cholangiocarcinoma	FTD/TPI + IRI	Efficacy/safety study (Germany)	Restarted

Abbreviations: 1L, first line; 2L, second line; 3L, third-line; BEV, bevacizumab; CRC, colorectal cancer; FTD/TPI, trifluridine/tipiracil; GC, gastric cancer; GEJC, gastroesophageal junction carcinoma; GI, gastrointestinal; ID, identifier; IRI, irinotecan; mCRC, metastatic colorectal cancer; N/A, not applicable; wt, wild type.

In three small studies in 2L+/3L+ mCRC, the triplet combination of BEV, FTD/TPI, and chemotherapy (OXA or IRI) resulted in DCRs exceeding 80% and median PFS > 6 months.^32,40,41^ Although preliminary, these data support further investigation of this triplet combination in previously treated mCRC.

In contrast to data in 2L+ and 3L+ mCRC, FTD/TPI + BEV did not improve outcomes compared with standard of care in untreated mCRC.^48^ In the noncomparative phase II TASCO1 study, promising clinical activity and tolerability was noted with FTD/TPI + BEV^48,50,55^; however, preliminary results from the ongoing comparative phase III SOLSTICE trial of FTD/TPI + BEV versus capecitabine + BEV in previously untreated mCRC indicated that FTD/TPI + BEV was not superior to capecitabine + BEV in the 1L setting; median PFS was similar with both regimens (9.3 vs. 9.4 months; HR 0.87, 95% CI, 0.75-1.02).^56^

Studies evaluating FTD/TPI in combination with targeted agents other than BEV were less common^21,30,35,44^ and among these, encouraging results were observed with FTD/TPI + PAN, an epidermal growth factor receptor (EGFR) antibody, with a median PFS of ≈ 6 months and median OS of ≈ 14 months.^44^ Ongoing phase II/III studies are evaluating FTD/TPI + PAN in both 1L (NCT05007132 EudraCT: 2019-004223-20) and 3L+ settings (NCT05468892) in mCRC (Table 4). While results from other studies included in this analysis were less conclusive,^21,30,51^ the combination of FTD/TPI with non-BEV targeted therapies is an area of active research and ongoing studies are exploring FTD/TPI in combination with agents targeting EGFR, MEK, VEGFR, and HER2 receptors (Table 4).

Other FTD/TPI-containing combinations were less effective and are not being pursued in phase II/III studies. Although phase I studies showed that FTD/TPI combined with OXA or IRI were tolerable in 2L+/3L+ mCRC, preliminary activity reported in 3 studies of FTD/TPI + OXA was not favorable (median PFS, ≈2 months; ORR, 0%-4%).^26,39,42^ Efficacy data with FTD/TPI + IRI are largely lacking or inconclusive.^19^ Similarly, combining immunotherapeutic agents with FTD/TPI has not proven to be efficacious: phase II studies of FTD/TPI + NIVO^45^ and FTD/TPI + NIVO + OXA^32^ were prematurely halted because of lack of efficacy.

Our analysis identified few published studies of FTD/TPI combinations tumor types other than CRC, such as gastroesophageal cancers, which is not surprising given that FTD/TPI monotherapy was only granted approval for this indication in 2019.^6^ Following the trend seen in mCRC, newly initiated or ongoing phases I and II studies in mGC/GEJC are exploring several FTD/TPI-containing combinations. Preliminary data from the phase III randomized Danish LonGas trial (EudraCT: 2018-004845-18; Table 4) indicated that adding BEV to FTD/TPI did not improve efficacy outcomes compared with FTD/TPI monotherapy for patients with pretreated metastatic esophagogastric adenocarcinoma, although OS and PFS benefits were seen with both regimens (median PFS, 3.7-3.9 months; OS, 9.0-9.9 months).^57^

While FTD/TPI combinations in other tumor types are being explored, such as two prospective studies of FTD/TPI + irinotecan in pretreated cholangiocarcinoma (Table 4), these efforts remain relatively rare.

The safety profiles of FTD/TPI-containing combinations mostly showed a higher incidence of grade ≥ 3 hematologic AEs. Neutropenia was the most common grade ≥ 3 AE associated with FTD/TPI-containing combinations, with varying incidence across different regimens and studies. Our analysis indicated that grade ≥ 3 neutropenia may occur in up to 72% of patients in the 2L+ setting,^7,8,46^ and up to 50% of patients in the 3L+ setting receiving FTD/TPI in combination with BEV.^28,29,37,38,43,47^ However, while neutropenia was more common with this combination than with FTD/TPI monotherapy,^8,38^ the difference was not statistically significant,^38^ and overall, treatment-related discontinuations were low in patients receiving FTD/TPI + BEV. Neutropenia has shown a dose-response relationship with FTD exposure, and higher FTD/TPI plasma levels are associated with improved OS and PFS, and a reduced time to performance status deterioration.^36,58^ In multiple studies, the presence of FTD/TPI-induced neutropenia—particularly high-grade neutropenia during early cycles—has been shown to be a useful predictive marker for clinical response and survival. ^36,58-60^

The incidence of non-hematologic grade ≥ 3 events remained low across studies in this analysis. Even in the recently reported phase III SOLSTICE trial, although the incidence of neutropenia was higher with the FTD/TPI + BEV compared with capecitabine + BEV, the non-hematologic safety profile of the former was more favorable.^56^ Importantly, cardiotoxicity was not a concern with FTD/TPI-containing combinations, unlike with fluoropyrimidines.^61^ In phase III studies, the overall incidence of grade ≥ 3 cardiac events was rare^4,5^ and a recent meta-analysis reported no increased cardiotoxicity risk with FTD/TPI compared with placebo.^62^ These data lend support for FTD/TPI as a drug of choice or as a good backbone for combination therapeutic regimens for patients with cardiac disease or cardiac side effects from previous chemotherapeutic regimens.^63^ Another patient subset that may benefit from FTD/TPI are those with dihydropyrimidine dehydrogenase (DPD) deficiency, as these patients are at risk of severe life-threatening AEs with 5-FU-containing regimens.^64^ As FTD/TPI is not metabolized by DPD,^65^ FTD/TPI is presumably safe for these patients. However, evidence of FTD/TPI’s safety in patients with DPD deficiency is limited to a few case reports^66-68^ and needs further clinical evaluation.

Quality-of-life (QoL) data were generally not reported in most studies included in this analysis, as these were early feasibility studies. One exception was the TASCO1 study of FTD/TPI + BEV in 1L mCRC. In this study, QoL was generally maintained during treatment, with no clinically relevant changes from baseline observed in global health status, functioning scales, and most symptom scales.^48^ Further data are needed to more fully establish the QoL impact of different FTD/TPI-based regimens, particularly among patients who were pretreated and/or over 65 years.

Limitations of this review were that the analysis captured phases I and II data only, with all but 2 studies being nonrandomized trials or retrospective observational studies. These studies were heterogeneous for combinations, treatment settings, and patient populations, which, in addition to the relatively small sample sizes (range, 9 to 97 patients), limited our ability to draw definite conclusions. Upcoming data from the phase III randomized SOLSTICE,^56^ SUNLIGHT (NCT04737187), and COLSTAR (NCT05223673) trials, which will comprise larger datasets, should help further elucidate the role of FTD/TPI combinations in mCRC. Data on non-BEV combinations in mCRC were also limited. This analysis lacked data from other tumor types, particularly mGC/GEJC; however, the bulk of these studies are underway or were recently reported, including the phase III study of FTD/TPI + bevacizumab in platinum-refractory mGC/GEJC (EudraCT: 2018-004845-18). Another possible limitation was that two-thirds (21/31) of the included studies were conducted in Japanese populations, highlighting the need for more data in diverse populations. Fortunately, a good number of ongoing FTD/TPI combination therapy trials are being conducted among populations in the US and Europe (Table 4).

Taken together, this comprehensive SLR consolidates the current body of evidence regarding FTD/TPI combinations in metastatic solid tumors and supports the feasibility and safety of certain FTD/TPI-containing combinations, such as FTD/TPI + BEV, in the guideline-recommended setting of refractory mCRC.

## Supplementary Material

Supplementary material is available at *The Oncologist* online.

oyae007_suppl_Supplementary_Material

## Data Availability

No new data were generated or analyzed in support of this research.
