# Supplementary material for: Efficacy and Safety of Trifluridine/Tipiracil-Containing Combinations in Colorectal Cancer and Other Advanced Solid Tumors: A Systematic Review
Source: Oncologist. 2024 Feb 16;29(5):e601–15. doi: 10.1093/oncolo/oyae007 (PMC11067808; doi:10.1093/oncolo/oyae007)
Supplement: oyae007_suppl_Supplementary_Material [file oyae007_suppl_supplementary_material.docx]

**Supporting information**

**Efficacy and safety of trifluridine/tipiracil-containing combinations in colorectal cancer and other advanced solid tumors: a systematic literature review**

**Short title:** Trifluridine/tipiracil-containing combinations in advanced solid tumors

**Authors**: Kohei Shitara,^a,b2*^ Alfred Falcone,^c^ Marwan G. Fakih,^d^ Ben George,^e^ Raghav Sundar,^f-h^ Sandip Ranjan,^i^ Eric Van Cutsem^j^

^a^National Cancer Center Hospital East, Chiba, Japan; ^b^Department of Immunology, Nagoya University Graduate School of Medicine, Nagoya, Japan; ^c^University of Pisa, Pisa, Italy; ^d^City of Hope Comprehensive Cancer Center, Duarte, CA, USA; ^e^Medical College of Wisconsin, Milwaukee, WI, USA; ^6^Department of Haematology-Oncology, National University Cancer Institute, Singapore, National University Hospital, Singapore; ^g^Cancer and Stem Cell Biology Program, Duke-NUS Medical School, Singapore; ^h^Yong Loo Lin School of Medicine, National University of Singapore, Singapore; ^i^SmartAnalyst, an Ashfield Advisory Company, Gurugram, Haryana, India; ^j^University Hospitals Gasthuisberg Leuven and KU Leuven, Leuven, Belgium

**Corresponding author:**

Kohei Shitara

Email: [kshitara@east.ncc.go.jp](mailto:kshitara@east.ncc.go.jp)

**Supplemental Online Table 1.** Search terms used for the systematic literature review (Medline)

| **#** | **Search strategy** |
| --- | --- |
| 1 | exp neoplasm/ or exp neoplasms/ |
| 2 | (cancer* or neoplasm* or tumo?r* or oncolog* or malignan* or carcinoma* or sarcoma* or adenocarcinoma* or leiomyosarcoma*).ti,ab,kw. |
| 3 | 1 or 2 |
| 4 | (lonsurf or tas 102 or tas102 or tas-102 or "trifluridine/tipiracil" or "Tipiracil-hydrochloride/trifluridine" or "Trifluridine/tipiracil hydrochloride" or "tipiracil hydrochloride plus trifluridine" or "trifluridine plus tipiracil" or "trifluridine plus tipiracil hydrochloride" or "tipiracil plus trifluridine" or "FTD/TPI" or "trifluridine - tipiracil" or "trifluridine-tipiracil" or ("trifluridine” and “tipiracil") or Orcantas or "S 95005" or "S 95005/TAS-102" or "T15, T20" or "T15/T20").ti,ab,kw. |
| 5 | 3 and 4 |
| 6 | limit 5 to humans |
| 7 | limit 6 to English language |

**Supplemental Online Table 2.** Search terms used for the systematic literature review (Embase)

| **#** | **Search strategy** |
| --- | --- |
| 1 | exp neoplasm/ or exp neoplasms/ |
| 2 | (cancer* or neoplasm* or tumo?r* or oncolog* or malignan* or carcinoma* or sarcoma* or adenocarcinoma* or leiomyosarcoma*).ti,ab,kw. |
| 3 | 1 or 2 |
| 4 | exp tipiracil plus trifluridine/ |
| 5 | (lonsurf or tas 102 or tas102 or tas-102 or "trifluridine/tipiracil" or "Tipiracil-hydrochloride/trifluridine" or "Trifluridine/tipiracil hydrochloride" or "tipiracil hydrochloride plus trifluridine" or "trifluridine plus tipiracil" or "trifluridine plus tipiracil hydrochloride" or "tipiracil plus trifluridine" or "FTD/TPI" or "trifluridine - tipiracil" or "trifluridine-tipiracil" or ("trifluridine" and "tipiracil") or Orcantas or "S 95005" or "S 95005/TAS-102" or "T15, T20" or "T15/T20").ti,ab,kw. |
| 6 | 4 or 5 |
| 7 | 3 and 6 |
| 8 | limit 7 to humans |
| 9 | limit 8 to English language |

**Supplemental Online Table 3.** Search terms used for the systematic literature review (Cochrane)

| **#** | **Search strategy** |
| --- | --- |
| 1 | [mh neoplasms] |
| 2 | (cancer* OR carcinoma* OR adenoma* OR adenocarcinoma* OR squamous* OR neoplas* OR tumor* OR tumour* OR malignan*):ti,ab,kw |
| 3 | 1 or 2 |
| 4 | (lonsurf OR "tas 102" OR tas102 OR "tas-102" OR "trifluridine/tipiracil" OR "Tipiracil-hydrochloride/trifluridine" OR "Trifluridine/tipiracil hydrochloride" OR "tipiracil hydrochloride plus trifluridine" OR "trifluridine plus tipiracil" OR "trifluridine plus tipiracil hydrochloride" OR "tipiracil plus trifluridine" OR "FTD/TPI" OR "trifluridine-tipiracil" OR ("trifluridine" and "tipiracil") OR Orcantas OR "S 95005" OR "S 95005/TAS-102" OR "T15, T20" OR "T15/T20"):ti,ab,kw |
| 5 | 3 and 4 |
| 6 | [mh humans] |
| 7 | ("Human" OR "Humans" OR "Homo sapiens" OR "Homo-sapiens" OR "Person" OR "Persons" OR "Human race" OR "Human-race" OR "Human being" OR "Human being" OR "Human-being" OR "Human-beings") |
| 8 | 6 or 7 |
| 9 | 5 and 8 |

**Supplemental Online Table 4.** Population, intervention, comparators, outcomes, and study design (PICOS) criteria employed in the systematic literature review

| **Category** | **Inclusion criteria** | **Exclusion criteria** |
| --- | --- | --- |
| Population | - Patients with cancer (any neoplasm) | - Nonhuman studies |
| Intervention | - FTD/TPI in combination with any other treatment | - Any study not including FTD/TPI in combination with other treatment |
| Comparators | - Any |  |
| Outcome | - Clinical efficacy: ORR, DCR, DOR, PFS, OS - Safety: Frequencies and grades of any AEs - Tolerability: Proportion of patients discontinuing treatment due to AEs - Quality of life | - Studies not reporting any of the relevant outcomes |
| Study design | - Randomized controlled trials - Non-randomized clinical trials - Observational studies | - Case studies, case reports, case series - Comments, editorials, narratives, letter to editor, opinion - Systematic literature reviews and meta-analyses will be excluded but earmarked for bibliographic search |
| Other criteria | - English language studies | - Non-English language studies |

Abbreviations: AE, adverse event; DCR, disease control rate; DOR, duration of response; FTD/TPI, trifluridine/tipiracil; ORR, overall response rate; OS, overall survival; PFS, progression-free survival**.**

**Supplemental Online Table 5.** OS with FTD/TPI + BEV in patients with metastatic colorectal cancer who were previously treated

| **Reference/ Study** | **Study type/phase** | **Patients with FTD/TPI + BEV, *n*** | **Treatment setting** | **Median OS  (95% CI)** | **HR (95% CI) vs FTD/TPI mono** | **12-month  OS rate, %** | **Median  follow-up (mo)** |
| --- | --- | --- | --- | --- | --- | --- | --- |
| FTD/TPI + BEV | | | | | | | |
| Pfeiffer 2020 [8] | Phase II RCT | 46 | 2L+ | 9.4 (7·6–10·7) | 0.55 (0.32–0.94) | **30** | 10·0 |
| Takahashi 2021 [46] | Phase II non-RCT | 97 | 2L+ | 9.1 (7.4–10.5) | NA | 35 | 15.8 |
| Kuboki 2017 [7] | Phase I/II non-RCT | 25 | 2L+ | 11·4 (7·6–13·9) | NA | 44 | 11·4 |
| Nose 2020 [36] | Retro Obs | 32 | 2L+ | 11.7 | 0.25 (0.13–0.48) | 50 | 11.6 |
| Fujii 2020 [33] | Retro Obs | 21 | 2L+ | 14.4 (7.9–NR) | 0.24 (0.12–0.52) | 89 | 14.8 |
| Hisamatsu 2019 [27] | Retro Obs | 24 | 2L+ | 12.7 (8.7–18.5) | NA | NA | NA |
| Ishikawa 2018 [22] | Retro Obs | 22 | 2L+ | 11.5 | NA | NA | NA |
| Makiyama 2018 [23] | Retro Obs | 11 | 2L+ | NR | 0.3 (0.09–0.99) | NA | NA |
| Ota 2016 [20] ^a^ | Retro Obs | 14 | 2L+ | 8.8 | NA | NA | 10.3 |
| Satake 2020 [37] | Phase Ib/II Non-RCT | 44 | 3L+ | 10.86 (8.32–13.68) | NA | 43 | 15.36 |
| Ishizaki 2021 [43] | Phase II Non-RCT | 19 | 3L+ | 11.5 (2.4–34.9) | NA | 59 | 11.5 |
| Yoshida 2021 [47] | Phase II Non-RCT | 32 | 3L+ | 9.2 (5.5–12.8) | NA | 37 | NA |
| Matsuhashi 2019 [29] | Retro Obs | 17 | 3L+ | 14.1 | NA | 99 | NA |
| Kotani 2019 [30] | Retro Obs | 60 | 3L+ | 8.6 (6.9–10.3) | 0.74 (0.48–1.14) | 38 | 7.1 |
| Yoshida 2018 [25] | Retro Obs | 25 | 3L+ | 13.2 | NA | 57 | NA |

^a^Data pertain to patients who received FTD/TPI ± BEV.

Abbreviations: 2L, second-line; 3L, third-line; BEV, bevacizumab; CI, confidence interval; FTD/TPI, trifluridine/tipiracil; HR, hazard ratio; mo, months; NA, not applicable; NR, not reached; OS, overall survival; RCT, randomized controlled trial; Retro Obs, retrospective observational.

**Supplemental Online Table 6.** PFS with FTD/TPI + BEV in patients with metastatic colorectal cancer who were previously treated

| **Reference/ Study** | **Study type/phase** | **Patients with FTD/TPI + BEV, *n*** | **Treatment setting** | **Median PFS  (95% CI)** | **HR (95% CI) vs FTD/TPI mono** | **6-month  PFS rate, %** | **Median  follow-up (mo)** |
| --- | --- | --- | --- | --- | --- | --- | --- |
| FTD/TPI + BEV | | | | | | | |
| Pfeiffer 2020 [8] | Phase II RCT | 46 | 2L+ | 4.6 (3·5–6·5) | 0·45 (0·29–0·72) | 43 | 10·0 |
| Takahashi 2021 [46] | Phase II non-RCT | 97 | 2L+ | 3.7 (2.6–4.1) | NA | 20 | 15.8 |
| Kuboki 2017^a^ [7] | Phase I/II non-RCT | 25 | 2L+ | 3.7 (2·0–5·4) | NA | 36 | 11·4 |
| Nose 2020 [36] | Retro Obs | 32 | 2L+ | 4.7 | 0.28 (0.15–0.51) | 34 | 11.6 |
| Hisamatsu 2019 [27] | Retro Obs | 24 | 2L+ | 5.4 (3.4–7.1) | NA | NA | NA |
| Ishikawa 2018 [22] | Retro Obs | 22 | 2L+ | 5.6 | NA | NA | NA |
| Makiyama 2018 [23] | Retro Obs | 11 | 2L+ | 5.8 | 0.34 (0.16–0.73) | NA | NA |
| Ota 2016^b^ [20] | Retro Obs | 14 | 2L+ | 4.7 | NA | NA | 10.3 |
| Miano 2020 [34] | Non-RCT | 15 | 3L+ | 4.8 (3.0–6.5) | NA | NA | 100 |
| Satake 2020 [37] | Phase Ib/II Non-RCT | 44 | 3L+ | 4.3 (2.5–5.8) | NA | 25 | 15.36 |
| Yoshida 2019 [31] | Phase II Non-RCT | 45 | 3L+ | 3.9 | NA | NA | NA |
| Ishizaki 2021 [43] | Phase II Non-RCT | 19 | 3L+ | 5.6 | NA | 42 | 11.5 |
| Yoshida 2021 [47] | Phase II Non-RCT | 32 | 3L+ | 4.5 (1.8–7.1) | NA | 27 | NA |
| Shibutani 2020 [38] | Retro Obs | 36 | 3L+ | 4.96 | NA | 29 | NA |
| Matsuhashi 2019 [29] | Retro Obs | 17 | 3L+ | 6.8 | NA | 52 | NA |
| Kotani 2019 [28] | Retro Obs | 60 | 3L+ | 3.7 (2.3–5.1) | 0.69 (0.48–0.99) | 23 | 7.1 |
| Yoshida 2018 [25] | Retro Obs | 25 | 3L+ | 4.2 | NA | 32 | NA |

^a^Median PFS was 3.7 by central assessment and 5.6 months by investigator assessment.

^b^Data pertain to patients receiving FTD/TPI ± BEV.

Abbreviations: 2L, second-line; 3L, third-line; BEV, bevacizumab; CI, confidence interval; FTD/TPI, trifluridine/tipiracil; HR, hazard ratio; mo, months; NA, not available; PFS, progression-free survival; RCT, randomized controlled trial; Retro Obs, retrospective observational.

**Supplemental Online Table 7.** OS with other FTD/TPI combinations in patients with metastatic colorectal cancer who were previously treated

| **Reference/ Study** | **Study type  and phase** | **Patients, *n*** | **Treatment setting** | **Intervention** | **Median OS  (95% CI)** | **12-month OS rate, %** | **Median  follow-up (mo)** |
| --- | --- | --- | --- | --- | --- | --- | --- |
| FTD/TPI + BEV + chemotherapy | | | | | | | |
| Bordonaro 2020 [32] | Phase I Non-RCT | 37 | 3L+ | FTD/TPI + BEV + OXA | 15.1 (10.7–NR) | NA | NA |
| FTD/TPI + chemotherapy | | | | | | | |
| Doi 2015 [19] | Phase I Non-RCT | 9 | 2L+ | FTD/TPI + IRI | 15.6 (7.5−NR) | NA | 33.7 |
| Cecchini 2021 [42] | Phase Ib/II Non-RCT | 41 | 3L+ | FTD/TPI + OXA | 6.8 (5.7–10) | 24 | 6.8 |
| Suenaga 2020 [39] | Phase I Non-RCT | 12 | 3L+ | FTD/TPI + OXA | 20.4 (9.2–31.6) | NA | 13.8 |
| FTD/TPI + targeted therapy | | | | | | | |
| Kato 2021 [44] | Phase I/II Non-RCT | 54 | 2L+ | FTD/TPI + PAN | 14.1 (12.2–19.3) | 66 | 16.5 |
| Yamazaki 2017 [21] | Phase I/II Non-RCT | 52 | 2L+ | FTD/TPI + NIN | 9.2 | NA | NA |
| Moehler 2020 [35] | Phase I Non-RCT | 12 | 3L+ | FTD/TPI + REG | NA | 50 | NA |

Abbreviations: 2L, second-line; 3L, third-line; BEV, bevacizumab; CI, confidence interval; FTD/TPI, trifluridine/tipiracil; IRI, irinotecan; mo, months; NA, not available; NIN, nintedanib; NR, not reached; OS, overall survival; OXA, oxaliplatin; PAN, panitumumab; RCT, randomized controlled trial; REG, regorafenib.

**Supplemental Online Table 8.** PFS with other FTD/TPI combinations in patients with metastatic colorectal cancer who were previously treated

| **Reference/ Study** | **Study type  and phase** | **Patients, *n*** | **Treatment setting** | **Intervention** | **Median PFS  (95% CI)** | **6-month PFS rate, %** | **Median  follow-up (mo)** |
| --- | --- | --- | --- | --- | --- | --- | --- |
| FTD/TPI + BEV + chemotherapy | | | | | | | |
| Varghese 2020 [40] | Phase 1 Non-RCT | 24 | 2L+ | FTD/TPI + BEV + IRI | 7.9 (5.1–13.4) | 66 | NA |
| Bordonaro 2020 [32] | Phase I Non-RCT | 37 | 3L+ | FTD/TPI + BEV + OXA | 6.3 (5.5–15.6) | 59 | NA |
|  |  | 17 |  | FTD/TPI + NIVO + OXA | 6 (2.0–8.0) | 61 | NA |
| FTD/TPI + chemotherapy | | | | | | | |
| Doi 2015 [19] | Phase I Non-RCT | 9 | 2L+ | FTD/TPI + IRI | 2.3 (1.9–6.2) | NA | 33.7 |
| Cecchini 2021 [42] | Phase Ib/II Non-RCT | 41 | 3L+ | FTD/TPI + OXA | 2.7 (2.4–4.8) | 26 | 6.8 |
| Suenaga 2020 [39] | Phase I Non-RCT | 12 | 3L+ | FTD/TPI + OXA | 2.4 (0.9–4.0) | NA | 13.8 |
| FTD/TPI + targeted therapy | | | | | | | |
| Kato 2021 [44] | Phase I/II Non-RCT | 54 | 2L+ | FTD/TPI + PAN | 5.8 (4.5–6.5) | 49 | 16.5 |
| Yamazaki 2017 [21] | Phase I/II Non-RCT | 52 | 2L+ | FTD/TPI + NIN | 3.7 | NA | NA |
| Moehler 2020 [35] | Phase I Non-RCT | 12 | 3L+ | FTD/TPI + REG | 3.8 (1.5–5.3) | NA | NA |
| Patel 2021 [45]^a^ | Phase II Non-RCT | 18 | 3L+ | FTD/TPI + NIVO | 2.8 (1.8–5.1) | 21 | NA |

^a^Median PFS was 2.2 months per immune-related response criteria and 2.8 months per response evaluation criteria in solid tumors (RECIST).

Abbreviations: 2L, second-line; 3L, third-line; BEV, bevacizumab; CI, confidence interval; FTD/TPI, trifluridine/tipiracil; IRI, irinotecan; mo, months NA, not applicable; NIN, nintedanib; NIVO, nivolumab; OXA, oxaliplatin; PAN, panitumumab; PFS, progression-free survival; RCT, randomized controlled trial; REG, regorafenib.

**Supplemental Online figure legends:**

**Supplemental Online Figure 1.** Objective response rates with **(A):** FTD/TPI + BEV and **(B):** FTD/TPI + other agents in patients with metastatic colorectal cancer who were previously treated.

Abbreviations: 2L+, second-line or later; 3L+, third-line or later; BEV, bevacizumab; chemo, chemotherapy; FTD/TPI, trifluridine/tipiracil; IRI, irinotecan; MUR, murlentamab; NA, not applicable; NIN, nintedanib; NIVO, nivolumab; NR, not reached; ORR, objective response rate; OXA, oxaliplatin; PAN, panitumumab; REG, regorafenib.


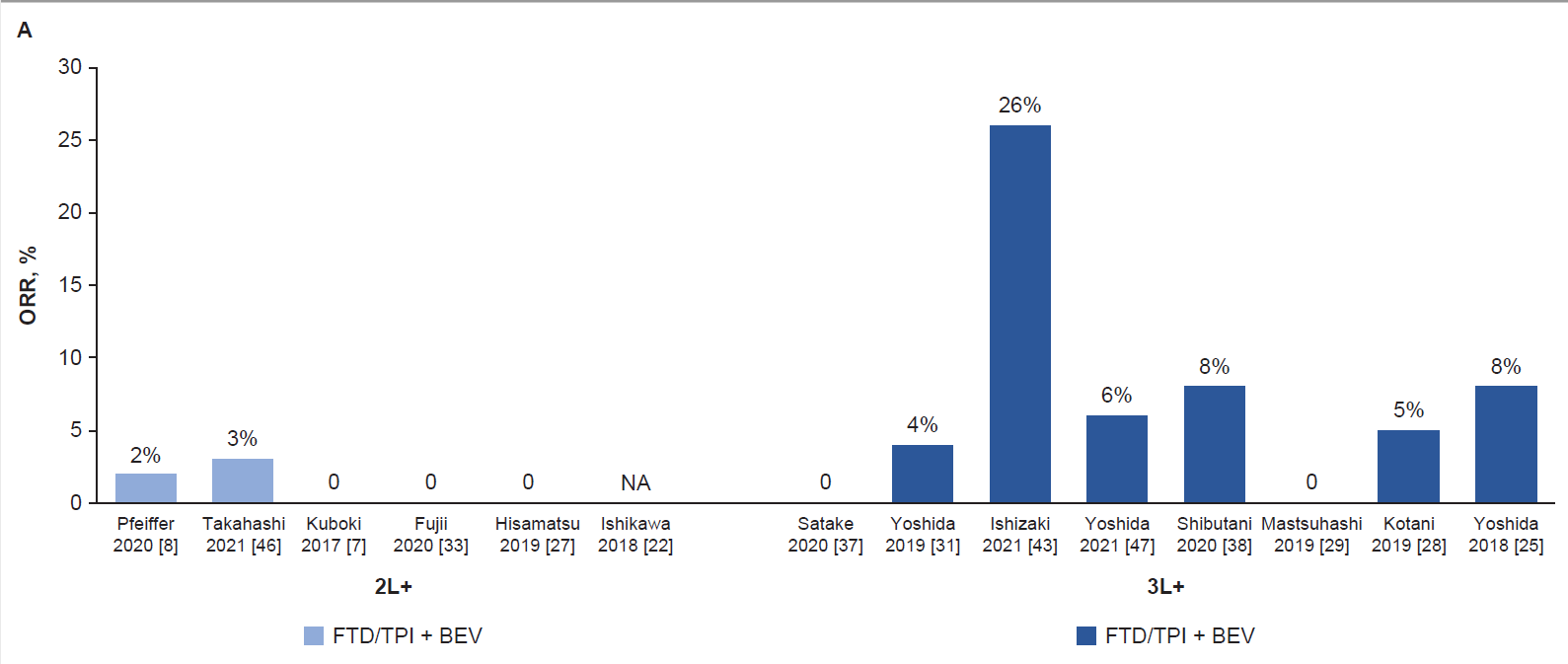


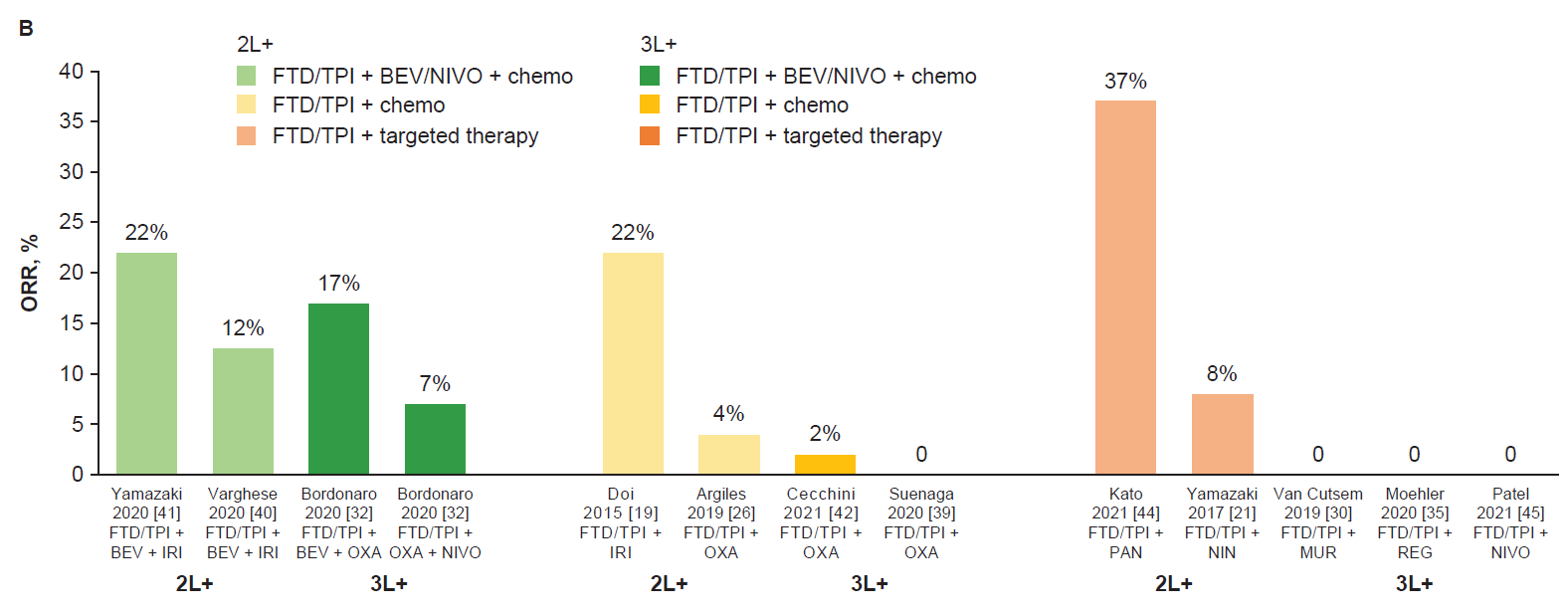


**Supplemental Online Figure 2.** Disease control rates with **(A):** FTD/TPI + BEV and **(B):** FTD/TPI + other agents in patients with metastatic colorectal cancer who were previously treated.

Abbreviations: 2L+, second-line or later; 3L+, third-line or later; BEV, bevacizumab; chemo, chemotherapy; DCR, disease control rate; FTD/TPI, trifluridine/tipiracil; IRI, irinotecan; MUR, murlentamab; NA, not applicable; NIN, nintedanib; NIVO, nivolumab; OXA, oxaliplatin; PAN, panitumumab; REG, regorafenib.


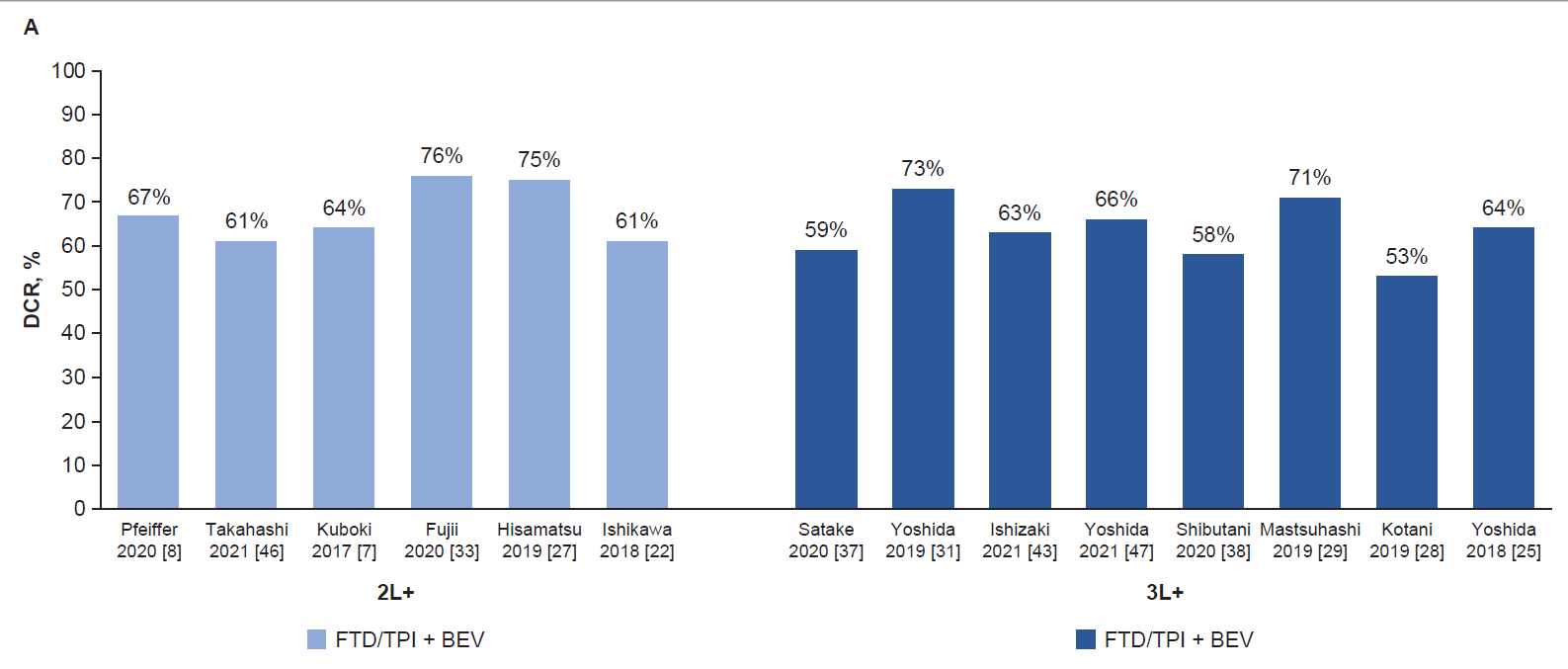


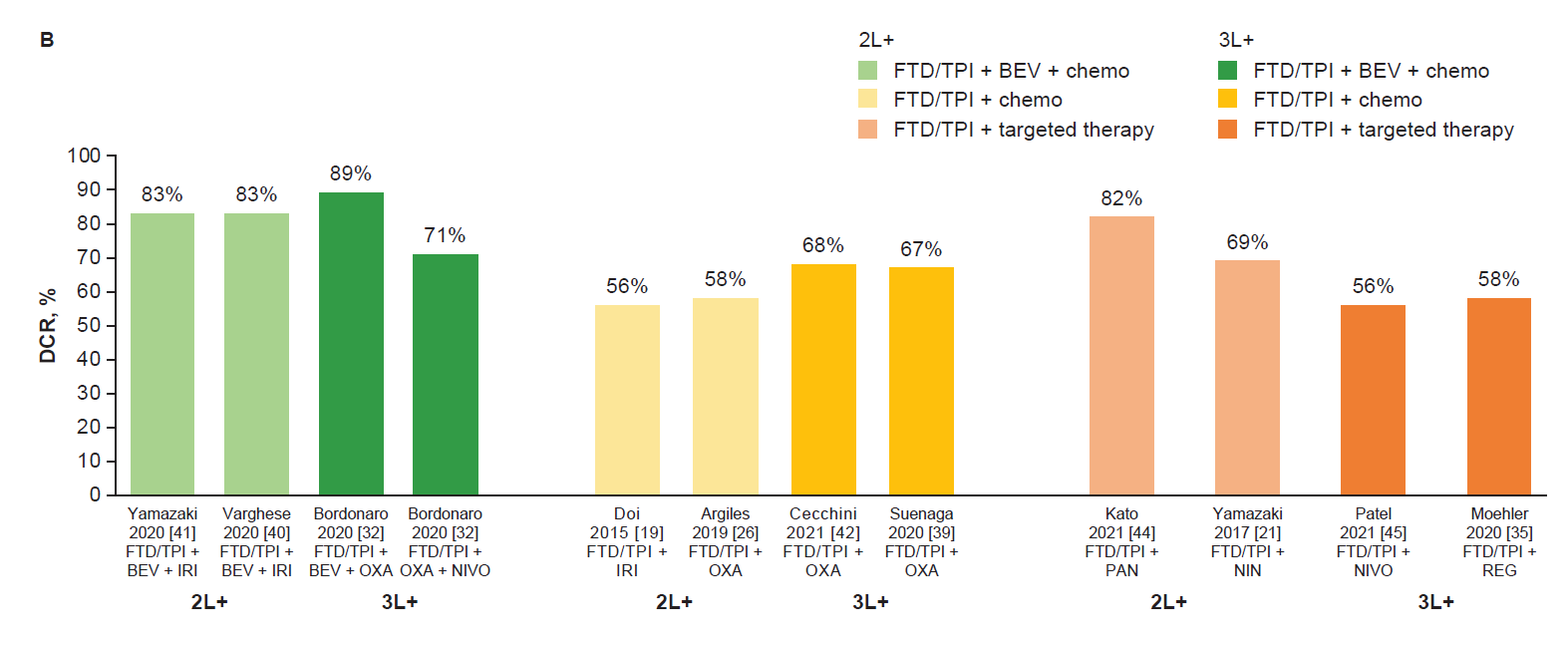


**REFERENCES**

1. Van Cutsem E, Cervantes A, Adam R et al. ESMO consensus guidelines for the management of patients with metastatic colorectal cancer. Ann Oncol 2016;27:1386–1422.

2. Peeters M, Cervantes A, Moreno Vera S et al. Trifluridine/tipiracil: an emerging strategy for the management of gastrointestinal cancers. Future Oncol 2018;14:1629–1645.

3. Emura T, Suzuki N, Yamaguchi M et al. A novel combination antimetabolite, TAS-102, exhibits antitumor activity in FU-resistant human cancer cells through a mechanism involving FTD incorporation in DNA. Int J Oncol 2004;25:571–578.

4. Mayer RJ, Van Cutsem E, Falcone A et al. Randomized trial of TAS-102 for refractory metastatic colorectal cancer. N Engl J Med 2015;372:1909–1919.

5. Shitara K, Doi T, Dvorkin M et al. Trifluridine/tipiracil versus placebo in patients with heavily pretreated metastatic gastric cancer (TAGS): a randomised, double-blind, placebo-controlled, phase 3 trial. Lancet Oncol 2018;19:1437–1448.

6. Drugs.com. Lonsurf FDA approval history.Available at: <https://www.drugs.com/history/lonsurf.html>. Accessed 15 July 2022.

7. Kuboki Y, Nishina T, Shinozaki E et al. TAS-102 plus bevacizumab for patients with metastatic colorectal cancer refractory to standard therapies (C-TASK FORCE): an investigator-initiated, open-label, single-arm, multicentre, phase 1/2 study. Lancet Oncol 2017;18:1172–1181.

8. Pfeiffer P, Yilmaz M, Möller S et al. TAS-102 with or without bevacizumab in patients with chemorefractory metastatic colorectal cancer: an investigator-initiated, open-label, randomised, phase 2 trial. Lancet Oncol 2020;21:412–420.

9. Referenced with permission from the NCCN Clinical Practice Guidelines in Oncology (NCCN Guidelines®) for Colon Cancer V.2.2022. ©National Comprehensive Cancer Network, Inc. 2022. All rights reserved. Accessed December 19, 2022.To view the most recent and complete version of the guideline, go online to NCCN.org. NCCN makes no warranties of any kind whatsoever regarding their content, use or application and disclaims any responsibility for their application or use in any way.

10. Referenced with permission from the NCCN Clinical Practice Guidelines in Oncology (NCCN Guidelines®) for Rectal Cancer V.3.2022. ©National Comprehensive Cancer Network, Inc. 2022. All rights reserved. Accessed December 19, 2022. To view the most recent and complete version of the guideline, go online to NCCN.org. NCCN makes no warranties of any kind whatsoever regarding their content, use or application and disclaims any responsibility for their application or use in any way.

11. Abrahao ABK, Ko Y-J, Berry S et al. A comparison of regorafenib and TAS-102 for metastatic colorectal cancer: a systematic review and network meta-analysis. Clin Colorectal Cancer 2018;17:113–120.

12. Sonbol MB, Benkhadra R, Wang Z et al. A systematic review and network meta-analysis of regorafenib and TAS-102 in refractory metastatic colorectal cancer. Oncologist 2019;24:1174–1179.

13. Andersen SE, Andersen IB, Jensen BV et al. A systematic review of observational studies of trifluridine/tipiracil (TAS-102) for metastatic colorectal cancer. Acta Oncol 2019;58:1149–1157.

14. Moher D, Liberati A, Tetzlaff J et al. Preferred reporting items for systematic reviews and meta-analyses: the PRISMA statement. PLoS Med 2009;6:e1000097.

15. Page MJ, McKenzie JE, Bossuyt PM et al. The PRISMA 2020 statement: an updated guideline for reporting systematic reviews. Rev Esp Cardiol (Engl Ed) 2021;74:790–799.

16. JPT Higgins, S Green, eds. *Cochrane Handbook for Systematic Reviews of Interventions. Version 5.1.0 (updated March 2011).* Oxford, U.K.: Cochrane Collaboration. 2011. Available at: <https://handbook-5-1.cochrane.org/>. Accessed 18 July 2022.

17. Downs SH, Black N. The feasibility of creating a checklist for the assessment of the methodological quality both of randomised and non-randomised studies of health care interventions. J Epidemiol Community Health 1998;52:377–384

18. Wells GA, Shea B, O’Connell D et al. The Newcastle-Ottawa Scale (NOS) for assessing the quality of nonrandomised studies in meta-analyses. Available at: <http://www.ohri.ca/programs/clinical_epidemiology/oxford.asp>. Accessed 18 July 2022.

19. Doi T, Yoshino T, Fuse N et al. Phase I study of TAS-102 and irinotecan combination therapy in Japanese patients with advanced colorectal cancer. Invest New Drugs 2015;33:1068–1677.

20. Ota T, Tsukuda H, Hasegawa Y et al. Treatment of TAS-102 in patients with metastatic colorectal cancer. Ann Oncol 2016;27(suppl_7):VII106–VII107.

21. Yamazaki K, Kuboki Y, Shinozaki E et al. A multicentre phase I/II study of TAS-102 with nintedanib in patients with metastatic colorectal cancer refractory to standard therapies (N-task force: EPOC1410). Ann Oncol 2017;28(suppl_5):V174–V175.

22. Ishikawa H, Suzuki K, Kakizawa N et al. Efficacy, tolerability, and manageability of TAS-102 plus bevacizumab for metastatic colorectal cancer patients in clinical practice. J Clin Oncol 2018;36(15_suppl):e15535.

23. Makiyama A, Yamaga S, Hirano G et al. A retrospective study to compare TAS-102 with TAS-102+BV in advanced colorectal cancer refractory to standard therapy. Ann Oncol 2018;29(Suppl_7):VII75.

24. Yasuda K, Kotani D, Kuboki Y et al. Safety for trifluridine/tipiracil (TAS-102) with bevacizumab combination in patients with refractory metastatic colorectal cancer in real-world clinical practice: the single-institutional experience. J Clin Oncol 2018;36(4_suppl):865–865.

25. Yoshida Y, Sakamoto R, Kajitani R et al. Biweekly administration of TAS-102 for neutropenia prevention in patients with colorectal cancer. Anticancer Res 2018;38:4367–4373.

26. Argilés G, André T, Hollebecque A et al. Phase I dose-escalation of trifluridine/tipiracil in combination with oxaliplatin in patients with metastatic colorectal cancer. Eur J Cancer 2019;112:12–19.

27. Hisamatsu A, Ikusue T, Toshima H et al. Retrospective study of TAS-102 plus bevacizumab for patients with metastatic colorectal cancer in salvage therapy. Ann Oncol 2019;30(suppl_4):IV93.

28. Kotani D, Kuboki Y, Horasawa S et al. Retrospective cohort study of trifluridine/tipiracil (TAS-102) plus bevacizumab versus trifluridine/tipiracil monotherapy for metastatic colorectal cancer. BMC Cancer 2019;19:1253.

29. Matsuhashi N, Takahashi T, Fujii H et al. Combination chemotherapy with TAS-102 plus bevacizumab in salvage-line treatment of metastatic colorectal cancer: a single-center, retrospective study examining the prognostic value of the modified Glasgow Prognostic Score in salvage-line therapy of metastatic colorectal cancer. Mol Clin Oncol 2019;11:390–396.

30. Van Cutsem E, Melichar B, Van den Eynde M et al. Phase 2 study results of murlentamab, a monoclonal antibody targeting the anti-mullerian-hormone-receptor II (AMHRII), acting through tumor-associated macrophage engagement in advanced/metastatic colorectal cancers. Ann Oncol 2019;30(suppl_4):IV153–IV154.

31. Yoshida Y, Yamada T, Matsuoka H et al. Biweekly TAS-102 and bevacizumab as a third-line chemotherapy for metastatic colorectal cancer: a phase II multicenter clinical trial (TAS-CC4 study). Ann Oncol 2019;30(suppl_5):v198–v252.

32. Bordonaro R, Calvo A, Auriemma A et al. Trifluridine/tipiracil in combination with oxaliplatin and either bevacizumab or nivolumab: results of the expansion part of a phase I study in patients with metastatic colorectal cancer. J Clin Oncol 2020;38(4_suppl):140–140.

33. Fujii H, Matsuhashi N, Kitahora Met al. Bevacizumab in combination with TAS-102 improves clinical outcomes in patients with refractory metastatic colorectal cancer: a retrospective study. Oncologist 2020;25:e469–e476.

34. Miano S, Francini G, Petrioli R et al. TAS-102 with bevacizumab in patients with chemorefractory metastatic colorectal cancer. Real fife study. Tumori Journal 2020;106(2_suppl):42–43.

35. Moehler M, Stein A, Trojan J et al. PD-8 Regorafenib with TAS-102 in metastatic colorectal cancer patients who progressed after at least two standard therapies: efficacy and safety results of a multicenter phase I study (REMETY). Ann Oncol 2020;31(suppl_3):S214.

36. Nose Y, Kagawa Y, Hata T et al. Neutropenia is an indicator of outcomes in metastatic colorectal cancer patients treated with FTD/TPI plus bevacizumab: a retrospective study. Cancer Chemother Pharmacol 2020;86:427–433.

37. Satake H, Kato T, Oba K et al. Phase Ib/II study of biweekly TAS-102 in combination with bevacizumab for patients with metastatic colorectal cancer refractory to standard therapies (BiTS study). Oncologist 2020;25:e1855–e1863.

38. Shibutani M, Nagahara H, Fukuoka T et al. Combining bevacizumab with trifluridine/thymidine phosphorylase inhibitor improves the survival outcomes regardless of the usage history of bevacizumab in front-line treatment of patients with metastatic colorectal cancer. Anticancer Res 2020;40:4157–4163.

39. Suenaga M, Wakatsuki T, Mashima T et al. A phase I study to determine the maximum tolerated dose of trifluridine/tipiracil and oxaliplatin in patients with refractory metastatic colorectal cancer: LUPIN study. Invest New Drugs 2020;38:111–119.

40. Varghese AM, Cardin DB, Hersch J et al. Phase I study of trifluridine/tipiracil plus irinotecan and bevacizumab in advanced gastrointestinal tumors. Clin Cancer Res 2020;26:1555–1562.

41. Yamazaki K, Masuishi T, Tsushima T et al. Phase Ib study of irinotecan, bevacizumab and biweekly trifluridine/tipiracil in patients with metastatic colorectal cancer refractory to fluoropyrimidine and oxaliplatin: preliminary report of MODURATE study. Ann Oncol 2020;31(suppl_3):S205.

42. Cecchini M, Kortmansky JS, Cui C et al. A phase 1b expansion study of TAS-102 with oxaliplatin for refractory metastatic colorectal cancer. Cancer 2021;127:1417–1424.

43. Ishizaki T, Mazaki J, Enomoto M et al. Prospective multicenter phase II study of biweekly TAS-102 and bevacizumab for metastatic colorectal cancer. Anticancer Res 2021;41:2157–2163.

44. Kato T, Kagawa Y, Kuboki Y et al. Safety and efficacy of panitumumab in combination with trifluridine/tipiracil for pre-treated patients with unresectable, metastatic colorectal cancer with wild-type RAS: The phase 1/2 APOLLON study. Int J Clin Oncol 2021;26:1238–1247.

45. Patel MR, Falchook GS, Hamada K et al. A phase 2 trial of trifluridine/tipiracil plus nivolumab in patients with heavily pretreated microsatellite-stable metastatic colorectal cancer. Cancer Med 2021;10:1183–1190.

46. Takahashi T, Yamazaki K, Oki E et al. Phase II study of trifluridine/tipiracil plus bevacizumab by RAS mutation status in patients with metastatic colorectal cancer refractory to standard therapies: JFMC51-1702-C7. ESMO Open 2021;6:100093.

47. Yoshida Y, Yamada T, Kamiyama H et al. Combination of TAS-102 and bevacizumab as third-line treatment for metastatic colorectal cancer: TAS-CC3 study. Int J Clin Oncol 2021;26:111–117.

48. Van Cutsem E, Danielewicz I, Saunders MP et al. Trifluridine/tipiracil plus bevacizumab in patients with untreated metastatic colorectal cancer ineligible for intensive therapy: the randomized TASCO1 study. Ann Oncol 2020;31:1160–1168.

49. Oki E, Makiyama A, Miyamoto Y et al. Trifluridine/tipiracil plus bevacizumab as a first-line treatment for elderly patients with metastatic colorectal cancer (KSCC1602): A multicenter phase II trial. Cancer Med 2021;10:454–461.

50. Van Cutsem E, Danielewicz I, Saunders MP et al. Phase II study evaluating trifluridine/tipiracil + bevacizumab and capecitabine + bevacizumab in first-line unresectable metastatic colorectal cancer (mCRC) patients who are noneligible for intensive therapy (TASCO1): Results of the final analysis on the overall survival. J Clin Oncol 2021;39(3_suppl):14–14.

51. Kasper S, Hofheinz RD, Stintzing S et al. 438 P Interim safety analysis of the phase IIb study of ramucirumab in combination with TAS102 vs. TAS102 monotherapy in metastatic colorectal cancer: The RAMTAS trial of the German AIO. Ann Oncol 2020;31(suppl_4):S427–S428.

52. Hara H, Mizukami T, Minashi K et al. A phase I/II trial of trifluridine/tipiracil in combination with irinotecan in patients with advanced gastric cancer refractory to fluoropyrimidine, platinum, and taxane. J Clin Oncol 2021;39(3_suppl):210–210.

53. Kawazoe A, Ando T, Hosaka H et al. Safety and activity of trifluridine/tipiracil and ramucirumab in previously treated advanced gastric cancer: an open-label, single-arm, phase 2 trial. Lancet Gastroenterol Hepatol 2021;6:209–217.

54. Uboha NV, Lubner SJ, LoConte NK et al. Phase 1 dose escalation trial of TAS-102 (trifluridine/tipiracil) and temozolomide in the treatment of advanced neuroendocrine tumors. Invest New Drugs 2020;38:1520–1525.

55. Van Cutsem E, Danielewicz I, Saunders MP et al. First-line trifluridine/tipiracil + bevacizumab in patients with unresectable metastatic colorectal cancer: final survival analysis in the TASCO1 study. Br J Cancer 2022;126:1548–1554.

56. André T, Falcone A, Shparyk Y et al. VP11-2021: Trifluridine/tipiracil plus bevacizumab vs capecitabine plus bevacizumab as first line treatment for patients with metastatic colorectal cancer (mCRC) ineligible for intensive therapy: The phase III randomized SOLSTICE study. Ann Oncol 2021;33229–230. [doi.org/10.1016/j.annonc.2021.11.006](https://doi.org/10.1016/j.annonc.2021.11.006)

57. Pfeiffer P, Yilmaz M, Nordsmark M et al. O-4 Trifluridine/tipiracil (TAS-102) with or without bevacizumab in patients with pretreated metastatic esophago-gastric adenocarcinoma (mEGA): A Danish randomized trial (LonGas). Ann Oncol. 2022;33(suppl_4):S380.

58. Yoshino T, Cleary JM, Van Cutsem E et al. Neutropenia and survival outcomes in metastatic colorectal cancer patients treated with trifluridine/tipiracil in the RECOURSE and J003 trials. Ann Oncol 2020;31:88–95.

59. Giuliani J, Bonetti A. The onset of frade ≥3 neutropenia is associated with longer overall survival in metastatic colorectal cancer patients treated with trifluridine/tipiracil. Anticancer Res 2019;39:3967–3969.

60. Kasi PM, Kotani D, Cecchini M et al. Chemotherapy induced neutropenia at 1-month mark is a predictor of overall survival in patients receiving TAS-102 for refractory metastatic colorectal cancer: a cohort study. BMC Cancer 2016;16:467.

61. Shiga T, Hiraide M. Cardiotoxicities of 5-fluorouracil and other fluoropyrimidines. Curr Treat Options Oncol 2020;21:27.

62. Lopez CA, Azimi-Nekoo E, Chung SY et al. Meta-analysis and systematic review of the cardiotoxicity of TAS-102. J Clin Oncol 2020;38(15_suppl):e16053-e16053.

63. Vaflard P, Ederhy S, Torregrosa C et al. [Fluoropyrimidines cardiac toxicity: 5-fluorouracil, capecitabine, compound S-1 and trifluridine/tipiracil]. Bull Cancer 2018;105:707–719.

64. Wörmann B, Bokemeyer C, Burmeister T et al. Dihydropyrimidine dehydrogenase testing prior to treatment with 5-fluorouracil, capecitabine, and tegafur: a consensus paper. Oncol Res Treat 2020;43:628–636.

65. Lenz H-J, Stintzing S, Loupakis F. TAS-102, a novel antitumor agent: a review of the mechanism of action. Cancer Treat Rev 2015;41:777–783.

66. Nakanishi R, Tsutsui A, Tanaka H et al. A case in which TAS-102 produced disease control without severe adverse events in a patient with recurrent colorectal cancer and dihydropyrimidine dehydrogenase deficiency. Med Clin Res 2021;6:726–729.

67. Schouten JF, Willems J, Sanders SJWJ et al. Standard-dose trifluridine/tipiracil as safe treatment alternative in metastatic colorectal cancer patients with DPD deficiency. Clin Colorectal Cancer 2021;20:359–363.

68. Bolzacchini E, Luchena G, Giordano M. Safety report of TAS-102 in a patient with reduced DPD activity. Clin Colorectal Cancer 2019;18:310–312.
